# Identification of Optically Active Pyrimidine Derivatives as Selective 5-HT_2C_ Modulators

**DOI:** 10.3390/molecules22091416

**Published:** 2017-08-26

**Authors:** Juhyeon Kim, Hanbyeol Jo, Hyunseung Lee, Hyunah Choo, Hak Joong Kim, Ae Nim Pae, Yong Seo Cho, Sun-Joon Min

**Affiliations:** 1 Center for Neuro-Medicine, Korea Institute of Science and Technology (KIST), 5 Hwarangno 14-gil, Seongbuk-gu, Seoul 02792, Korea; wngus0307@nate.com (J.K.); hchoo@kist.re.kr (H.C.); 2 Department of Chemistry, Korea University, Seoul 02841, Korea; hakkim@korea.ac.kr; 3 Department of Chemical & Molecular Engineering/Applied Chemistry, Hanyang University, Ansan, Gyeonggi-do 15588, Korea; haim_e@naver.com (H.J.); lhs9087@naver.com (H.L.); 4 Department of Biological Chemistry, Korea University of Science and Technology (UST), 217 Gajungro, Yuseong-gu, Daejeon 34113, Korea; anpae@kist.re.kr; 5 Convergence Research Center for Diagnosis, Treatment and Care System of Dementia, KIST, Seoul 02792, Korea

**Keywords:** pyrimidine, optically active, enzymatic kinetic resolution, 5-HT_2C_ receptor, binding affinity, selectivity

## Abstract

A series of pyrimidine derivatives **4a**–**i** were synthesized and evaluated for their binding affinities towards 5-HT_2C_ receptors. With regard to designed molecules **4a**–**i**, the influence of the size of alkyl ether and the absolute configuration of a stereogenic center on the 5-HT_2C_ binding affinity and selectivity was studied. The most promising diasteromeric mixtures **4d** and **4e** were selected in the initial radioligand binding assay and they were further synthesized as optically active forms starting from optically active alcohols **5d** and **5e**, prepared by an enzymatic kinetic resolution. Pyrimidine analogue (*R,R*)-**4e** displayed an excellent 5-HT_2C_ binding affinity with good selectivity values against a broad range of other 5-HT receptor subtypes.

## 1. Introduction

Serotonin receptors, also known as 5-hydroxytryptamine (5-HT) receptors, are mainly located in the central nervous system (CNS) and play an important role in mediating both excitatory and inhibitory neurotransmission. There are 14 different 5-HT receptors classified into seven major subfamilies (5-HT_1–7_). They have been known to regulate various physiological functions such as mood, depressive behavior, appetite, biorhythm, and feeding [1,2]. According to recent studies, the 5-HT_2C_ receptor (5-HT_2C_R) is expected to be a potential drug target for the diagnosis and treatment of a number of CNS disorders including schizophrenia, depression, substance abuse, and Parkinson diseases, as well as obesity and urinary incontinence [3,4,5,6,7,8]. In particular, 5-HT_2C_-specific modulators may have few undesired side effects on peripheral tissues because this receptor is exclusively expressed in the CNS [9,10]. However, the 5-HT_2C_ receptor belongs to the 5-HT_2_ receptor family, together with 5-HT_2A_ and 5-HT_2B_, which have a high similarity in terms of their amino acid sequences [11]. It has been reported that the activation of 5-HT_2A_ and 5-HT_2B_ is strongly implicated in hallucinations and valvular heart disease [12,13]. Thus, the discovery of 5-HT_2C_ agonists with a high specificity and subtype selectivity for 5-HT_2A_ and 5-HT_2B_ receptors is important for avoiding side effects.

**Figure 1:**
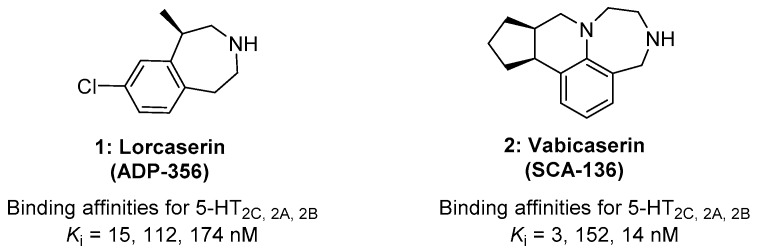
The chemical structures of representative 5-HT_2C_ receptor ligands and their binding affinities for 5-HT_2_ receptor subtypes [19,21].

To date, several compounds, including lorcaserin **1** and vabicaserin **2**, have been identified as 5-HT_2C_ agonists with significant selectivity against 5-HT_2A_ and 5-HT_2B_ (Figure 1) [14,15,16,17,18]. Lorcaserin (ADP-356) was developed for the treatment of obesity and was approved for clinical use by FDA in 2012 [19]. Vabicaserin is a drug developed for the treatment of acute schizophrenia or appetite suppressants, but has not been shown to be effective in clinical trial studies [20,21]. In addition, other small molecule 5-HT_2C_ agonists have been reported to be in preclinical or early clinical development [15,16,17,18].

**Figure 2:**
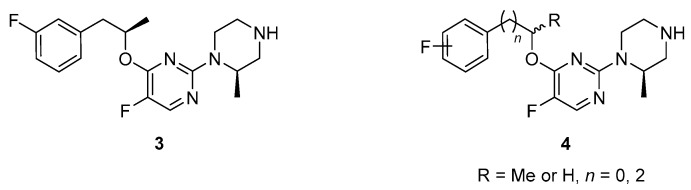
The structures of reported 5-HT_2C_ agonist **3** and designed compounds **4**.

During our efforts toward the development of 5-HT receptor modulators, we have initiated a program to discover 5-HT_2C_ agonists as potential therapeutic and diagnostic agents for CNS diseases. Recently, pyrimidine analogue **3** with good pharmacological and pharmacokinetic properties has been reported as a potential 5-HT_2C_ agonist [22]. Our in vitro study of this molecule proved that it has a high potency against the 5-HT_2C_ receptor and relative good selectivity against the 5-HT_2A_ receptor [23]. However, the binding affinity of **3** to the 5-HT_2B_ receptor is still too high, which prompted us to investigate this compound for the development of 5-HT_2C_ selective agonists. Based on the report of the activation selectivity of 5-HT_2C_ over 5-HT_2B_ [11], we postulated that altering the chain length between the phenyl group and pyrimidine may induce a subtle structural change in the molecule, which can make a large difference in its interaction with 5-HT_2C_ and 5-HT_2B_ because the binding site of 5-HT_2C_ is slightly deeper than that of 5-HT_2B_ [11]. Thus, pyrimidine derivatives **4** with hydrocarbon chains shorter or longer than that of the parent molecule **3** were designed to examine the effect of the structural modification of compound **3** on the target selectivity toward 5-HT_2C_ over 5-HT_2B_ (Figure 2). In this paper, we report our progress in the synthesis and biological evaluation of pyrimidine derivatives **4** as selective 5-HT_2C_ agonists.

## 2. Results and Discussion

### 2.1. Synthesis of Pyrimidine Derivatives

**Scheme 1:**
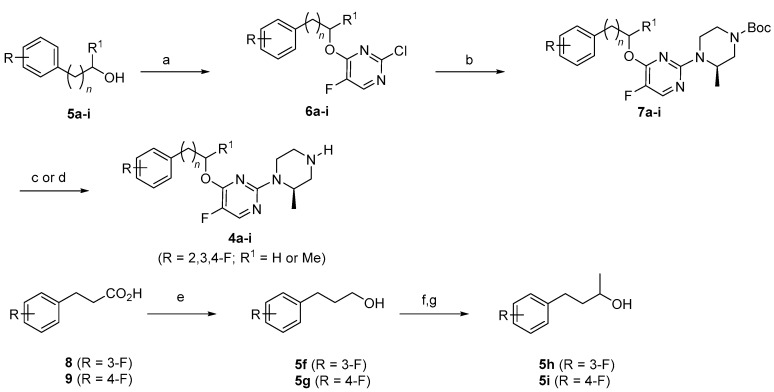
Synthesis of the pyrimidine derivatives **4a**–**i**. Reagents and conditions: (**a**) 2,4-dichloro-5-fluoropyrimidine, NaO*t*Bu, toluene, 0 °C, 70–91%; (**b**) (*R*)-(+)-1-Boc-3-methylpiperazine, DIPEA, CH_3_CN, 110 °C, 14–53%; (**c**) TFA, CH_2_Cl_2_, r.t., 52–72%; (**d**) 4 M HCl, dioxane, 0 °C, 53%; (**e**) BH_3_-Me_2_S, THF, r.t., 84–94%; (**f**) PCC, CH_2_Cl_2_, r.t., 73–77%; (**g**) MeMgBr, THF, 0 °C, 72–85%.

The synthesis of compounds **4a**–**i** is described in Scheme 1. Following the literature procedure [22,24], a series of pyrimidine derivatives **4a**–**i** were synthesized starting from either primary or secondary alcohols **5a**–**i**. The alcohols **5a**–**e** were obtained from commercial sources, whereas the others **5f**–**i** were easily prepared via the reduction/oxidation of phenyl propanoic acids **8** and **9**. Thus, alcohols **5a**–**i** were first reacted with 2,4-dichloro-5-fluoropyrimidine in the presence of NaO*t*Bu to afford 4-alkoxypyrmidines **6a**–**i**. A nucleophilic aromatic substitution reaction (S_N_Ar) of **6a**–**i** with (*R*)-(+)-1-Boc-3-methylpiperazine gave 2-amino-4-alkoxypiperazine **7a**–**i**. Finally, the BOC group in **7a**–**i** was removed in the presence of trifluoroacetic acid or 4 M hydrochloric acid to furnish the corresponding pyrimidine derivatives **4a**–**i**.

### 2.2. Biological Evaluation

The serotonin receptor binding affinity of our synthesized compounds **4a**–**i** was examined by a radioligand binding assay in transfected CHO-K1 cell lines using [^3^H]mesulergine as a radioligand. Practically, a displacement of radioligand with compounds **4a**–**i** was first evaluated at a concentration of 10 μM, and then their *K*_i_ values were determined on the basis of the dose-response curves. As summarized in Table 1, compounds **4d** and **4e** showed the highest binding affinities to 5-HT_2C_ and good selectivity values for 5-HT_2A_. However, the binding affinities of **4d** and **4e** to 5-HT_2B_ were comparable to the value of **3**. At this moment, we assumed that both diastereomeric mixtures **4d** and **4e** might have a negative influence on the selectivity for 5-HT_2B_. Thus, we planned to synthesize each diastereomer derived from **4d** and **4e** as an optically pure form to test its in vitro activity against 5-HT_2_ receptors.

### 2.3. Synthesis and In Vitro Evaluation of Optically Active Pyrimidines

**Scheme 2:**
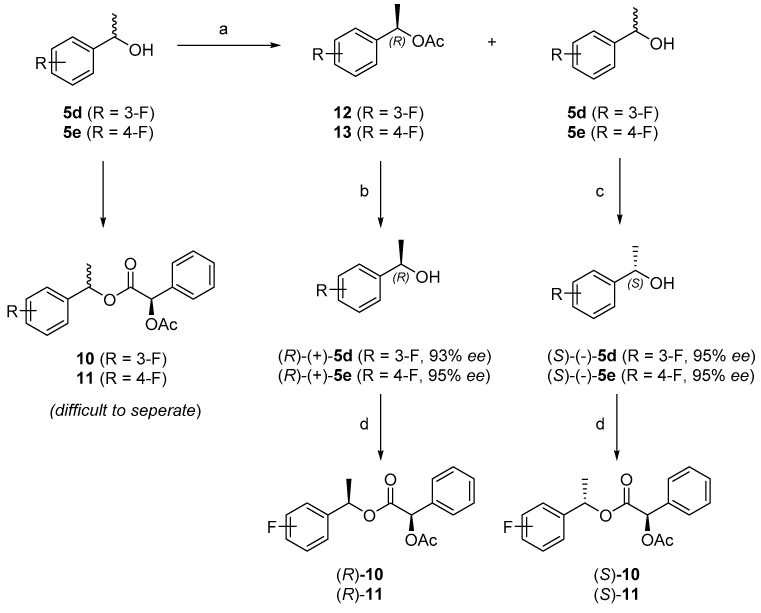
The synthesis of (*R*)/(*S*)-**5d** and **5e** using enzymatic kinetic resolution. Reagents and conditions: (**a**) vinyl acetate (0.5 eq), CAL-B, pyridine, hexane, r.t.; (**b**) 1 M NaOH, MeOH, r.t., 17–32% (two steps); (**c**) vinyl acetate (1 eq), CAL-B, pyridine, hexane, r.t., then separation, 44–46%; (**d**) (*R*)-(−)-*O*-acetylmadelic acid, DCC, DMAP, CH_2_Cl_2_, r.t., 68–86%.

In order to synthesize **4d** and **4e** as optically active diastereomers, optically active secondary alcohols **5d** and **5e** should be prepared. For this purpose, we initially attempted the separation of diastereomers **10** and **11**, which were synthesized by the reaction of **5d** and **5e** with (*R*)-(−)-*O*-acetylmadelic acid using EDCI as a coupling reagent. However, diastereomeric mixtures **10** and **11** were not completely separated by column chromatography on silica gel. Alternatively, the enzymatic kinetic resolution was applied to separate racemic **5d** and **5e,** as shown in Scheme 2. It has been reported that CAL-B lipase can selectively acetylate the (*R*)-form of secondary benzyl alcohols using vinyl acetate as an acyl transfer reagent (Scheme 2) [25,26]. According to the literature procedure, the selective acetylation reactions of racemic secondary alcohols **5d** and **5e** were performed using 0.5 equivalent of vinyl acetate, pyridine, and the CAL-B enzyme in hexane. Enantiomeric pure acetate **12** and **13** were separated and then hydrolyzed to afford the desired (*R*)-forms of **5d** and **5e**. It should be noted that we obtained each diasteromeric **5d** and **5e** with a high optical purity when hexane was used as a solvent, although an ionic liquid such as [bmim][PF_6_] and [bmim][BF_4_] was used to enhance the enantiomeric selectivity of lipases in the literature. Additionally, (*S*)-**5d** and (*S*)-**5e** were successfully obtained by further acetylation of the remaining alcohols **5d** and **5e** with an excess of vinyl acetate and CAL-B enzyme followed by chromatographic separation.

For determining the optical purity of (*R*)/(*S*)-**5d** and **5e**, they were converted to the corresponding mandelic ester (*R*)/(*S*)-**10** and **11**, respectively. A ^1^H-NMR analysis of diastereomeric protons in (*R*)/(*S*)-**10** and **11** indicated that the *ee*’s of (*R*/(*S*)-**5d** and **5e** were greater than 93%. We also confirmed that the optical rotation values of our compounds (*R*/(*S*)-**5d** and **5e** are almost identical to those of the compounds reported in the literature.

**Scheme 3:**
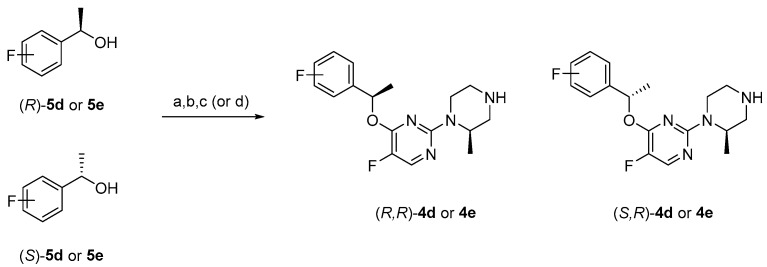
Synthesis of the optically active pyrimidine derivatives **4d**–**4e**. Reagents and conditions: (**a**) 2,4-dichloro-5-fluoropyrimidine, NaO*t*Bu, toluene, 0 °C, 55–72%; (**b**) (*R*)-(+)-1-Boc-3-methylpiperazine, toluene, 150 °C, 44–57%; (**c**) TFA, CH_2_Cl_2_, r.t., 67–71%; (**d**) HCl, dioxane, 0 °C, 81–82%.

With each enantiomer (*R*)- or (*S*)-**5d** and **5e** in hand, the optically active pyrimidine derivatives **4d**–**4e** were synthesized following the same reaction sequences (Scheme 3). Finally, compounds **4d** and **4e** were assessed for their binding affinity to 5-HT_2_ receptor subtypes by radioligand binding assays. The in vitro assay results are demonstrated in Table 2. In general, (*R,R*)-**4d** and **4e** prepared from secondary alcohols (*R*)-**5d** and (*R*)-**5e** showed excellent binding affinities to 5-HT_2C_, whereas (*S*,*R*)-forms of **4d** and **4e** exhibited low potencies for 5-HT_2A_ and 5-HT_2B_. A further evaluation of these compounds for other 5-HT receptor subtypes was also performed and is provided in the supplementary data (Table S2). These results combined with the in vitro data in Table 1 suggested that pyrimidine derivatives **4** with a short alkyl chain could maintain their binding affinity to 5-HT_2C_ comparable to that of **3** and the binding affinity for other 5-HT subtypes could be significantly influenced by an absolute configuration of the stereogenic methyl group in **4**. Considering the potencies and selectivities of **4**, we can conclude that (*R*,*R*)-**4e** would be a viable candidate for a selective 5-HT_2C_ modulator.

## 3. Materials and Methods

### 3.1. General Methods

All reactions were conducted under oven-dried glassware under an atmosphere of nitrogen. All commercially available reagents were purchased and used without further purification. Solvents and gases were dried according to standard procedures. Organic solvents were evaporated with reduced pressure using a rotary evaporator. Reactions were followed by analytical thin layer chromatography (TLC) analysis using glass plates precoated with silica gel (0.25 mm). TLC plates were visualized by exposure to UV light (UV), and were then visualized with a KMnO_4_ or *p*-anisaldehyde stain followed by brief heating on a hot plate. Flash column chromatography was performed using silica gel 60 (230–400 mesh, Merck, Darmstadt, Germany) with the indicated solvents. ^1^H-NMR spectra were measured with 400MHz and ^13^C-NMR spectra were measured with 100MHz using CDCl_3_ and MeOD. ^1^H-NMR spectra are represented as follows: chemical shift, multiplicity (s = singlet, d = doublet, t = triplet, q = quartet, m = multiplet), integration, and coupling constant (*J*) in Hertz (Hz). ^1^H-NMR chemical shifts are reported relative to CDCl_3_ (7.26 ppm). ^13^C NMR was recorded relative to the central line of CDCl_3_ (77.0 ppm). High resolution mass spectra (HR-MS) were obtained using positive electrospray ionization and mass/charge (*m/z*) ratios that are reported as values in atomic mass units.

### 3.2. Synthesis of Pyrimidines ***4a**–**i***

#### 3.2.1. General Procedure for Preparing Compounds **5f** and **5g**

To a solution of 3-(3 or 4-fluorophenyl)propanoic acid **8** or **9** (1.78 mmol) in THF (8.90 mL), borane-dimethyl sulfide (3.57 mmol) was added dropwise. The reaction mixture was allowed to stir at room temperature for 1 h. After completion of the reaction (monitored by TLC), the mixture was slowly quenched with MeOH until bubbling ceased, extracted with EtOAc. The organic layers were dried over anhydrous MgSO_4_ and concentrated in vacuo. The resulting residue was purified by flash column chromatography on silica gel (EtOAc:*n*-hexane = 1:4) to afford propanol **5f** or **5g**.

*3-(3-Fluorophenyl)propan-1-ol* (**5f**): Yield: 84%; ^1^H-NMR (400 MHz, CDCl_3_) *δ* 7.26–7.21 (m, 1H), 6.97 (d, *J* = 7.6 Hz, 1H), 6.92–6.86 (m, 2H), 3.68 (q, *J* = 5.8 Hz, 2H), 2.72 (t, *J* = 7.7 Hz, 2H), 1.92–1.85 (m, 2H). ^13^C-NMR (100 MHz, CDCl_3_) *δ* 162.9 (d, ^1^*J* = 243 Hz), 144.4 (d, ^3^*J* = 7 Hz), 129.8 (d, ^3^*J* = 8 Hz), 124.1 (d, ^4^*J* = 3 Hz), 115.2 (d, ^2^*J* = 21 Hz), 112.7 (d, ^2^*J* = 21 Hz), 62.0, 33.9, 31.7 (d, ^4^*J* = 2 Hz).

*3-(4-Fluorophenyl)propan-1-ol* (**5g**): Yield: 94%; ^1^H-NMR (400 MHz, CDCl_3_) *δ* 7.17–7.14 (m, 2H), 7.00–6.95 (m, 2H), 3.67 (t, *J* = 6.4 Hz, 2H), 2.69 (t, *J* = 7.8 Hz, 2H), 3.67 (t, *J* = 6.4 Hz, 2H), 1.91–1.84 (m, 2H). ^13^C-NMR (100 MHz, CDCl_3_) *δ* 161.6 (d, ^1^*J* = 241.6 Hz), 137.7 (d, ^4^*J* = 3.1 Hz), 130.0 (d, ^3^*J* = 7.6 Hz), 115.4 (d, ^2^*J* = 21 Hz), 62.3, 34.6, 31.5.

#### 3.2.2. General Procedure for Preparing Compounds **5f’** and **5g’**

To a solution of propanol **5f** or **5g** (0.482 mmol) in CH_2_Cl_2_ (4.80 mL), PCC (0.964 mmol) was added at 0 °C. The reaction mixture was allowed to stir at room temperature for 1 h. After completion of the reaction (monitored by TLC), the mixture was filtered with silica gel and a celite pad, and extracted with ether. The organic layers were dried over anhydrous MgSO_4_ and concentrated in vacuo. The resulting residue was purified by flash column chromatography on silica gel (EtOAc:*n*-hexane = 1:4) to afford propanal **5f’** or **5g’**.

*3-(3-Fluorophenyl)propanal* (**5f’**): Yield: 77%; ^1^H-NMR (400 MHz, CDCl_3_) *δ* 9.82 (t, *J* = 1.2 Hz, 1H), 7.27–7.22 (m, 1H), 6.97 (d, *J* = 7.8 Hz, 1H), 6.91–6.88 (m, 2H), 2.95 (t, *J* = 7.5 Hz, 2H), 2.80–2.77 (m, 2H). ^13^C-NMR (100 MHz, CDCl_3_) *δ* 201.0, 162.9 (d, ^1^*J* = 242 Hz), 142.9 (d, ^3^*J* = 8 Hz), 130.4 (d, ^3^*J* = 8 Hz), 124.0 (d, ^4^*J* = 2 Hz), 115.2 (d, ^2^*J* = 21 Hz), 113.2 (d, ^2^*J* = 21 Hz), 44.9, 27.8 (d, ^4^*J* = 2 Hz).

*3-(4-Fluorophenyl)propanal* (**5g’**): Yield: 73%; ^1^H-NMR (400 MHz, CDCl_3_) *δ* 9.77 (t, *J* = 1.2 Hz, 1H), 7.13–7.09 (m, 2H), 6.96–6.91 (m, 2H), 2.91–2.87 (m, 2H), 2.72 (t, *J* = 7.5 Hz, 2H). ^13^C-NMR (100 MHz, CDCl_3_) *δ* 201.2, 161.5 (d, ^1^*J* = 243 Hz), 136.0 (d, ^4^*J* = 3 Hz), 129.7 (d, ^3^*J* = 8 Hz), 115.3 (d, ^2^*J* = 21 Hz), 45.4, 27.3.

#### 3.2.3. General Procedure for Preparing Compounds **5h** and **5i**

To a solution of propanal **5f’** or **5g’** (0.204 mmol) in THF (2.00 mL), MeMgBr (3.0 M in ether: 0.245 mmol) was added dropwise at 0 °C. The reaction mixture was allowed to stir at room temperature for 1 h. After completion of the reaction (monitored by TLC), it was quenched with saturated aqueous NH_4_Cl, extracted with EtOAc and washed with brine. The organic layers were dried over anhydrous MgSO_4_ and concentrated in vacuo. The resulting residue was purified by flash column chromatography on silica gel (EtOAc:*n*-hexane = 1:2) to afford butan-2-ol **5h** or **5i**.

*4-(3-Fluorophenyl)butan-2-ol* (**5h**): Yield: 85%; ^1^H-NMR (400 MHz, CDCl_3_) *δ* 7.26–7.20 (m, 1H), 6.97 (d, *J* = 7.7 Hz, 1H), 6.92–6.85 (m, 2H), 3.82 (d, *J* = 2.3 Hz, 1H), 2.80–2.63 (m, 2H), 1.79–1.73 (m, 2H), 1.24 (d, *J* = 6.2 Hz, 3H).

*4-(4-Fluorophenyl)butan-2-ol* (**5i**): Yield: 72%; ^1^H-NMR (400 MHz, CDCl_3_) *δ* 7.12 (dd, *J* = 5.6, 8.2 Hz, 2H), 6.93 (t, *J* = 8.7 Hz, 2H), 3.82–3.75 (m, 1H), 2.74–2.58 (m, 2H), 1.75–1.68 (m, 2H), 1.20 (d, *J* = 6.2 Hz, 3H). ^13^C-NMR (100 MHz, CDCl_3_) *δ* 161.3 (d, ^1^*J* = 241 Hz), 137.7 (d, ^4^*J* = 3 Hz), 129.7 (d, ^3^*J* = 8 Hz), 115.1 (d, ^2^*J* = 21 Hz), 67.4, 40.9, 31.3, 23.7.

#### 3.2.4. General Procedure for Preparing Compounds **6a**–**i**

A solution of sodium *tert*-butoxide (2.72 mmol) in toluene (18.2 mL) was treated with primary or secondary alcohol (1.82 mmol) dropwise at 0 °C. After 5 min, 2,4-dichloro-5-fluoropyrimidine (2.18 mmol) was added to the mixture. The reaction mixture was allowed to stir at room temperature for 1 h. After completion of the reaction (monitored by TLC), it was quenched with saturated aqueous NH_4_Cl, extracted with EtOAc, and washed with brine. The organic layers were dried over anhydrous MgSO_4_ and concentrated in vacuo. The resulting residue was purified by flash column chromatography on silica gel (EtOAc:*n*-hexane = 1:8) to afford pyrimidine **6**.

*2-Chloro-5-fluoro-4-((2-fluorobenzyl)oxy)pyrimidine* (**6a**): Yield: 91%; ^1^H-NMR (400 MHz, CDCl_3_) *δ* 8.21 (d, *J* = 2.2 Hz, 1H), 7.53–7.49 (m, 1H), 7.49–7.34 (m, 1H), 7.20–7.09 (m, 2H), 5.57 (s, 2H). ^13^C-NMR (100 MHz, CDCl_3_) *δ* 161.2 (d, ^1^*J* = 247 Hz), 159.0 (d, ^2^*J* = 12 Hz), 153.2 (d, ^4^*J* = 5 Hz), 145.9 (d, ^1^*J* = 263 Hz), 144.3 (d, ^2^*J* = 20 Hz), 131.1 (d, ^3^*J* = 4 Hz), 131.0 (d, ^3^*J* = 8 Hz), 124.3 (d, ^4^*J* = 4 Hz), 121.8 (d, ^2^*J* = 14 Hz), 115.7 (d, ^2^*J* = 20 Hz), 63.9 (d, ^3^*J* = 4 Hz).

*2-Chloro-5-fluoro-4-((3-fluorobenzyl)oxy)pyrimidine* (**6b**): Yield: 90%; ^1^H-NMR (400 MHz, CDCl_3_) *δ* 8.23 (d, *J* = 2.2 Hz, 1H), 7.40–7.34 (m, 1H), 7.25 (d, *J* = 7.26 Hz, 1H), 7.19 (d, *J* = 9.4 Hz, 1H), 7.09–7.04 (m, 1H), 5.50 (s, 2H). ^13^C-NMR (100 MHz, CDCl_3_) *δ* 162.9 (d, ^1^*J* = 245 Hz), 158.9 (d, ^2^*J* = 11 Hz), 153.2 (d, ^4^*J* = 5 Hz), 145.8 (d, ^1^*J* = 245 Hz), 144.4 (d, ^2^*J* = 20 Hz), 136.9 (d, ^3^*J* = 8 Hz), 130.3 (d, ^3^*J* = 8 Hz), 123.9 (d, ^4^*J* = 3 Hz), 115.7 (d, ^2^*J* = 21 Hz), 115.3 (d, ^2^*J* = 22 Hz), 68.9 (d, ^4^*J* = 2 Hz).

*2-Chloro-5-fluoro-4-((4-fluorobenzyl)oxy)pyrimidine* (**6c**): Yield: 75%; ^1^H-NMR (400 MHz, CDCl_3_) *δ* 8.20 (d, *J* = 2.2 Hz, 1H), 7.47 (dd, *J* = 5.4 Hz, 8.7Hz, 2H), 7.08 (t, *J* = 8.7 Hz, 2H), 5.47 (s, 2H). ^13^C-NMR (100 MHz, CDCl_3_) *δ* 163 (d, ^1^*J* = 246 Hz), 159.03 (d, ^2^*J* = 12 Hz), 153.2 (d, ^4^*J* = 5 Hz), 145 (d, ^1^*J* = 263 Hz), 144.3 (d, ^2^*J* = 20 Hz), 130.8 (d, ^3^*J* = 9 Hz), 130.3 (d, ^3^*J* = 4 Hz), 115.2 (d, ^2^*J* = 21 Hz), 69.2.

*2-Chloro-5-fluoro-4-(1-(3-fluorophenyl)ethoxy)pyrimidine* (**6d**): Yield: 79%; ^1^H-NMR (400 MHz, CDCl_3_) *δ* 8.17 (d, *J* = 2.2 Hz, 1H), 7.35–7.30 (m, 1H), 7.22 (d, *J* = 7.7 Hz, 1H), 7.17–7.14 (m, 1H), 7.02–6.97 (m, 1H), 6.31–6.27 (m, 1H), 1.70 (d, *J* = 6.6 Hz, 3H). ^13^C-NMR (100 MHz, CDCl_3_) *δ* 162.9 (d, ^1^*J* = 245 Hz), 158.6 (d, ^2^*J* = 11 Hz), 153.2 (d, ^4^*J* = 5 Hz), 146.0 (d, ^1^*J* = 263 Hz), 144.4 (d, ^2^*J* = 20 Hz), 142.9 (d, ^3^*J* = 7 Hz), 130.2 (d, ^3^*J* = 8 Hz), 122.0 (d, ^4^*J* = 2 Hz), 115.3 (d, ^2^*J* = 21 Hz), 113.3 (d, ^2^*J* = 22 Hz), 75.7 (d, ^4^*J* = 2 Hz), 22.2.

*2-Chloro-5-fluoro-4-(1-(4-fluorophenyl)ethoxy)pyrimidine* (**6e**): Yield: 89%; ^1^H-NMR (400 MHz, CDCl_3_) *δ* 8.35 (d, *J* = 2.2 Hz, 1H), 7.66 (dd, *J* = 5.3, 8.7 Hz, 2H), 7.29–7.25 (m, 2H), 6.54–6.49 (m, 1H), 1.92 (d, *J* = 6.6 Hz, 3H). ^13^C-NMR (100 MHz, CDCl_3_) *δ* 162.6 (d, ^1^*J* = 245 Hz), 158.6 (d, ^2^*J* = 11 Hz), 153.1 (d, ^4^*J* = 5 Hz), 146.0 (d, ^1^*J* = 262 Hz), 144.3 (d, ^2^*J* = 20 Hz), 136.1 (d, ^4^*J* = 3 Hz), 128.4 (d, ^3^*J* = 8 Hz), 115.6 (d, ^2^*J* = 22 Hz), 75.9, 22.1.

*2-Chloro-5-fluoro-4-(3-(3-fluorophenyl)propoxy)pyrimidine* (**6f**): Yield: 86%; ^1^H-NMR (400 MHz, CDCl_3_) *δ* 8.19 (d, *J* = 2.2 Hz, 1H), 7.28–7.22 (m, 1H), 6.98 (d, *J* = 7.7 Hz, 1H), 6.92–6.87 (m, 2H), 4.48 (t, *J* = 6.4 Hz, 2H), 2.80 (t, *J* = 7.6 Hz, 2H), 2.19–2.12 (m, 2H). ^13^C-NMR (100 MHz, CDCl_3_) *δ* 163.0 (d, ^1^*J* = 244 Hz), 159.4 (d, ^2^*J* = 11 Hz), 153.3 (d, ^4^*J* = 5 Hz), 145.9 (d, ^1^*J* = 263 Hz), 144.0 (d, ^2^*J* = 20 Hz), 143.3 (d, ^3^*J* = 7 Hz), 123.0 (d, ^3^*J* = 8 Hz), 124.1 (d, ^4^*J* = 3 Hz), 115.3 (d, ^2^*J* = 21 Hz), 113.1 (d, ^2^*J* = 21 Hz), 67.6, 31.6 (d, ^4^*J* = 1 Hz), 29.6.

*2-Chloro-5-fluoro-4-(3-(4-fluorophenyl)propoxy)pyrimidine* (**6g**): Yield: 93%; ^1^H-NMR (400 MHz, CDCl_3_) *δ* 8.17 (d, *J* = 4.2 Hz, 1H), 7.16–7.12 (m, 2H), 6.99–6.93 (m, 2H), 4.45 (t, *J* = 6.4 Hz, 2H), 2.75 (t, *J* = 7.6 Hz, 2H), 2.14–2.10 (m, 2H). ^13^C-NMR (100 MHz, CDCl_3_) *δ* 161.4 (d, ^1^*J* = 246 Hz), 159.4 (d, ^2^*J* = 11 Hz), 153.3 (d, ^4^*J* = 5 Hz), 145.9 (d, ^1^*J* = 262 Hz), 144.0 (d, ^2^*J* = 20 Hz), 136.3 (d, ^4^*J* = 3 Hz), 129.8 (d, ^3^*J* = 8 Hz), 115.3 (d, ^2^*J* = 21 Hz), 67.6, 31.1, 30.0.

*2-Chloro-5-fluoro-4-((4-(3-fluorophenyl)butan-2-yl)oxy)pyrimidine* (**6h**): Yield: 70%; ^1^H-NMR (400 MHz, CDCl_3_) *δ* 8.17 (d, *J* = 2.3 Hz, 1H), 7.26–7.20 (m, 1H), 6.95–6.87 (m, 3H), 5.40–5.35 (m, 1H), 2.79–2.69 (m, 2H), 2.18–2.11 (m, 1H), 2.02–1.96 (m, 1H), 1.43 (d, *J* = 6.2 Hz, 3H). ^13^C-NMR (100 MHz, CDCl_3_) *δ* 163.0 (d, ^1^*J* = 244 Hz), 159.2 (d, ^2^*J* = 11 Hz), 153.3 (d, ^4^*J* = 5 Hz), 146.0 (d, ^1^*J* = 262 Hz), 144.1 (d, ^2^*J* = 20 Hz), 143.6 (d, ^3^*J* = 7 Hz), 129.9 (d, ^3^*J* = 8 Hz), 124.0 (d, ^4^*J* = 3 Hz), 115.2 (d, ^2^*J* = 21 Hz), 113.0 (d, ^2^*J* = 21 Hz), 75.1, 37.0, 31.4 (d, ^4^*J* = 1 Hz), 19.6.

*2-Chloro-5-fluoro-4-((4-(4-fluorophenyl)butan-2-yl)oxy)pyrimidine* (**6i**): Yield: 79%; ^1^H-NMR (400 MHz, CDCl_3_) *δ* 8.17 (d, *J* = 2.3 Hz, 1H), 7.14–7.11 (m, 2H), 6.99–6.94 (m, 2H), 5.40–5.35 (m, 1H), 2.78–2.65 (m, 2H), 2.17–2.09 (m, 1H), 2.00–1.92 (m, 1H), 1.42 (d, *J* = 6.2 Hz, 3H). ^13^C-NMR (100 MHz, CDCl_3_) *δ* 161.4 (d, ^1^*J* = 242 Hz), 159.2 (d, ^2^*J* = 11 Hz), 153.3 (d, ^4^*J* = 5 Hz), 146.0 (d, ^1^*J* = 262 Hz), 144.0 (d, ^2^*J* = 21 Hz), 136.6 (d, ^4^*J* = 3 Hz), 129.7 (d, ^3^*J* = 8 Hz), 115.2 (d, ^2^*J* = 21 Hz), 75.2, 37.4, 30.8, 19.6.

#### 3.2.5. General Procedure for Preparing Compounds **7a**–**i**

To a solution of pyrimidine **6** (0.569 mmol) in CH_3_CN (2.90 mL), (*R*)-(+)-1-Boc-3-methylpiperazine (1.14 mmol) and *N*,*N*-diisopropylethylamine (1.14 mmol) were added. The reaction mixture was allowed to stir at 110 °C for 14 h. After completion of the reaction (monitored by TLC), it was quenched with saturated aqueous NH_4_Cl, extracted with EtOAc, and washed with brine. The organic layers were dried over anhydrous MgSO_4_ and concentrated in vacuo. The resulting residue was purified by flash column chromatography on silica gel (EtOAc/CHCl_3_/*n*-hexane = 1:4:8) to afford methyl piperazine carboxylate **7**.

*(R)-tert-Butyl-4-(5-fluoro-4-((2-fluorobenzyl)oxy)pyrimidin-2-yl)-3-methylpiperazine-1-carboxylate* (**7a**): Yield: 14%; ^1^H-NMR (400 MHz, CDCl_3_) *δ* 7.98 (d, *J* = 2.8 Hz, 1H), 7.49–7.45 (m, 1H), 7.35–7.29 (m, 1H), 7.17–7.13 (m, 1H), 7.11–7.06 (m, 1H), 5.53–5.44 (m, 2H), 4.73 (bs, 1H), 4.32 (d, *J* =13.3, 1H), 4.14–3.90 (m, 2H), 3.17–2.89 (m, 3H), 1.49 (s, 9H), 1.12 (d, *J* = 6.7 Hz, 3H). ^13^C-NMR (100 MHz, CDCl_3_) *δ* 160.7 (d, ^1^*J* = 246 Hz), 157.6 (d, ^2^*J* = 11 Hz), 156.8 (d, ^4^*J* = 2 Hz), 155.2, 143.5 (d, ^2^*J* = 19 Hz), 139.9 (d, ^1^*J* = 247 Hz), 130.08 (d, ^3^*J* = 4 Hz), 130.05 (d, ^3^*J* = 9 Hz), 124.2 (d, ^3^*J* = 3 Hz), 123.4 (d, ^2^*J* = 14 Hz), 115.4 (d, ^2^*J* = 21 Hz), 79.8, 67.6 (d, ^3^*J* = 5 Hz), 48.5, 47.2, 44.0, 42.9, 38.7, 28.4, 14.1. 

*(R)-tert-Butyl-4-(5-fluoro-4-((3-fluorobenzyl)oxy)pyrimidin-2-yl)-3-methylpiperazine-1-carboxylate* (**7b**): Yield: 27%; ^1^H-NMR (400 MHz, CDCl_3_) *δ* 7.99 (d, *J* = 2.8 Hz, 1H), 7.36–7.30 (m, 1H), 7.19 (d, *J* = 7.7 Hz, 1H), 7.15–7.09 (m, 1H), 7.03–6.98 (m, 1H), 5.43–5.36 (m, 2H), 4.69 (bs, 1H), 4.29 (d, *J* = 13.0 Hz, 1H), 4.14–3.84 (m, 2H), 3.16–2.87 (m, 3H), 1.48 (s, 9H), 1.11 (d, *J* = 6.6 Hz, 3H). ^13^C-NMR (100 MHz, CDCl_3_) *δ* 162.9 (d, ^1^*J* = 245 Hz), 157.5 (d, ^2^*J* = 10 Hz), 156.8 (d, ^4^*J* = 2 Hz), 155.2, 143.6 (d, ^2^*J* = 19 Hz), 139.9 (d, ^1^*J* = 240 Hz), 138.7 (d, ^3^*J* = 7 Hz), 130.1 (d, ^3^*J* = 8 Hz), 123.2 (d, ^4^*J* = 3 Hz), 115.1 (d, ^2^*J* = 21 Hz), 114.6 (d, ^2^*J* = 22 Hz), 79.9, 67.1 (d, ^4^*J* = 1 Hz), 48.5, 47.2, 44.0, 42.9, 38.8, 28.4, 14.1.

*(R)-tert-Butyl-4-(5-fluoro-4-((4-fluorobenzyl)oxy)pyrimidin-2-yl)-3-methylpiperazine-1-carboxylate* (**7c**): Yield: 21%; ^1^H-NMR (400 MHz, CDCl_3_) *δ* 7.98 (d, *J* = 2.8 Hz, 1H), 7.43–7.39 (m, 2H), 7.07–7.03 (m, 2H), 5.40–5.34 (m, 2H), 4.72 (bs, 1H), 4.30 (d, *J* = 12.8 Hz, 1H), 4.15–3.78 (m, 2H), 3.17–2.87 (m, 3H), 1.48 (s, 9H), 1.13 (d, *J* = 6.7 Hz, 3H). ^13^C-NMR (100 MHz, CDCl_3_) *δ* 162.7 (d, ^1^*J* = 245 Hz), 157.7 (d, ^2^*J* = 11 Hz), 156.8 (d, ^4^*J* = 2 Hz), 155.2, 143.5 (d, ^2^*J* = 20 Hz), 139.9 (d, ^1^*J* = 247 Hz), 131.9 (d, ^4^*J* = 3 Hz), 129.9 (d, ^3^*J* = 8 Hz), 115.5 (d, ^2^*J* = 21 Hz), 79.9, 67.2, 48.5, 47.2, 44.0, 42.9, 38.8, 28.4, 14.1.

*(R)-tert-Butyl-4-(5-fluoro-4-(1-(3-fluorophenyl)ethoxy)pyrimidin-2-yl)-3-methylpiperazine-1-carboxylate* (**7d**): Yield: 44%; ^1^H-NMR (400 MHz, CDCl_3_, Diastereomeric mixture) *δ* 7.94–7.93 (m, 1H, 1H′), 7.32–7.25 (m, 1H, 1H′), 7.16–7.06 (m, 2H, 2H′), 6.97–6.90 (m, 1H, 1H′), 6.09–6.00 (m, 1H, 1H′), 4.57 (bs, 1H, 1H′), 4.22–3.85 (m, 3H, 3H′), 3.08–2.74 (m, 3H, 3H′), 1.65 (d, *J* = 6.6 Hz, 3H, 3H′), 1.46 (d, *J* = 2.4 Hz, 9H, 9H′), 1.13–0.90 (m, 3H, 3H′). ^13^C-NMR (100 MHz, CDCl_3_, Diastereomeric mixture) *δ* 162.9 (d, ^1^*J* = 244 Hz), 157.14 (d, ^3^*J* = 11 Hz), 157.11 (d, ^3^*J* = 11 Hz), 156.7 (d, ^4^*J* = 3 Hz), 155.20, 155.18, 145.0 (d, ^2^*J* = 23 Hz), 144.91 (d, ^2^*J* = 23 Hz), 143.47 (d, ^2^*J* = 20 Hz), 143.44 (d, ^2^*J* = 20 Hz), 140.0 (d, ^1^*J* = 245 Hz), 130.11 (d, ^3^*J* = 8 Hz), 130.07 (d, ^3^*J* = 9 Hz), 121.42 (d, ^4^*J* = 3 Hz), 121.17 (d, ^4^*J* = 3 Hz), 114.6 (d, ^2^*J* = 21 Hz), 114.5 (d, ^2^*J* = 21 Hz), 112.8 (d, ^2^*J* = 22 Hz), 112.6 (d, ^2^*J* = 22 Hz), 79.8, 79.8, 73.92, 73.90, 73.80, 73.79, 48.4, 47.1, 44.0, 42.8, 38.7, 28.4, 23.0, 22.8, 13.9.

*(R)-tert-Butyl-4-(5-fluoro-4-(1-(4-fluorophenyl)ethoxy)pyrimidin-2-yl)-3-methylpiperazine-1-carboxylate* (**7e**): Yield: 40%; ^1^H-NMR (400 MHz, CDCl_3_, Diastereomeric mixture) *δ* 7.95–7.94 (m, 1H, 1H′), 7.94–7.33 (m, 2H, 2H′), 7.06–6.98 (m, 2H, 2H′), 6.12–6.05 (m, 1H, 1H′), 4.60 (bs, 1H, 1H′), 4.25–3.88 (m, 3H, 3H′), 3.10–2.87 (m, 3H, 3H′), 1.66 (d, *J* = 6.6 Hz, 3H, 3H′), 1.48 (d, *J* = 2.0 Hz, 9H, 9H′), 1.15–0.96 (m, 3H, 3H′). ^13^C-NMR (100 MHz, CDCl_3_, Diastereomeric mixture) *δ* 162.3 (d, ^1^*J* = 245 Hz), 162.2 (d, ^1^*J* = 245 Hz), 157.3 (d, ^2^*J* = 11 Hz), 157.2 (d, ^2^*J* = 11 Hz), 156.7, 155.2, 143.4 (d, ^2^*J* = 20 Hz), 143.3 (d, ^2^*J* = 19 Hz), 140.0 (d, ^1^*J* = 247 Hz), 138.1 (d, ^4^*J* = 3 Hz), 137.9 (d, ^4^*J* = 3 Hz), 127.7 (d, ^3^*J* = 8 Hz), 127.4 (d, ^3^*J* = 8 Hz), 115.5 (d, ^2^*J* = 22 Hz), 115.4 (d, ^2^*J* = 21 Hz), 79.9, 73.9, 73.8, 48.5, 47.2, 43.8, 42.9, 38.71, 38.68, 28.4, 23.0, 22.8, 14.0.

*(R)-tert-Butyl-4-(5-fluoro-4-(3-(3-fluorophenyl)propoxy)pyrimidin-2-yl)-3-methylpiperazine-1-carboxylate* (**7f**): Yield: 53%; ^1^H-NMR (400 MHz, CDCl_3_) *δ* 7.94 (d, *J* = 3.0 Hz, 1H), 7.26–7.20 (m, 1H), 6.96 (d, *J* = 7.7 Hz, 1H), 6.90–6.85 (m, 2H), 4.65 (bs, 1H), 4.35 (t, *J* = 6.5 Hz, 2H), 4.25–4.21 (m, 1H), 4.18–3.88 (m, 2H), 3.13–2.87 (m, 3H), 2.77 (t, *J* = 7.6 Hz, 2H), 2.13–2.06 (m, 2H), 1.47 (s, 9H), 1.12 (d, *J* = 6.7 Hz, 3H). ^13^C-NMR (100 MHz, CDCl_3_) *δ* 163.6 (d, ^1^*J* = 244 Hz), 158.7 (d, ^2^*J* = 11 Hz), 157.5 (d, ^4^*J* = 2 Hz), 155.9, 144.4 (d, ^3^*J* = 7 Hz), 143.8 (d, ^2^*J* = 20 Hz), 140.6 (d, ^1^*J* = 247 Hz), 130.5 (d, ^3^*J* = 9 Hz), 124.8 (d, ^4^*J* = 3 Hz), 115.9 (d, ^2^*J* = 21 Hz), 113.6 (d, ^2^*J* = 21 Hz), 80.4, 66.0, 49.1, 47.8, 44.6, 43.5, 39.4, 32.5, 30.6, 29.1, 14.6.

*(R)-tert-Butyl-4-(5-fluoro-4-(3-(4-fluorophenyl)propoxy)pyrimidin-2-yl)-3-methylpiperazine-1-carboxylate* (**7g**): Yield: 45%; ^1^H-NMR (400 MHz, CDCl_3_) *δ* 7.96 (d, *J* = 2.9 Hz, 1H), 7.16–7.13 (m, 2H), 6.99–6.94 (m, 2H), 4.66 (bs, 1H), 4.39–4.33 (m, 2H), 4.25–3.89 (m, 3H) 3.20–3.06 (m, 2H), 3.02–2.98 (m, 1H), 2.88–2.73 (m, 2H), 2.12–2.05 (m, 2H), 1.48 (s, 9H), 1.12 (d, *J* = 6.6 Hz, 3H). ^13^C-NMR (100 MHz, CDCl_3_) *δ* 161.4 (d, ^1^*J* = 242 Hz), 158.1 (d, ^2^*J* = 11 Hz), 156.9 (d, ^4^*J* = 3 Hz), 155.2, 143.1 (d, ^2^*J* = 20 Hz), 140.0 (d, ^1^*J* = 247 Hz), 136.8 (d, ^4^*J* = 3 Hz), 129.8 (d, ^3^*J* = 8 Hz), 115.2 (d, ^2^*J* = 21 Hz), 79.8, 65.4, 48.5, 47.1, 44.0, 42.9, 38.7, 31.3, 30.4, 28.4, 14.0.

*(R)-tert-Butyl-4-(5-fluoro-4-((4-(3-fluorophenyl)butan-2-yl)oxy)pyrimidin-2-yl)-3-methylpiperazine-1-carboxylate* (**7h**): Yield: 45%; ^1^H-NMR (400 MHz, CDCl_3_, Diastereomeric mixture) *δ* 7.96 (d, *J* = 2.9 Hz, 1H, 1H′), 7.24–7.18 (m, 1H, 1H′), 6.94–6.84 (m, 3H, 3H′), 5.27–5.19 (m, 1H, 1H′), 4.64–4.61 (m, 1H, 1H′), 4.22–3.88 (m, 3H, 3H′), 3.13–3.05 (m, 2H, 2H′), 2.88–2.67 (m, 3H, 3H′), 2.17–2.08 (m, 1H, 1H′), 1.96–1.87 (m, 1H, 1H′), 1.48 (d, *J* = 0.7 Hz, 9H, 9H′), 1.38 (d, *J* = 6.2 Hz, 3H, 3H′), 1.13–1.10 (m, 3H, 3H′). ^13^C-NMR (100 MHz, CDCl_3_, Diastereomeric mixture) *δ* 162.9 (d, ^1^*J* = 244 Hz), 157.8 (d, ^2^*J* = 11 Hz), 156.9 (d, ^4^*J* = 3 Hz), 155.2, 144.06 (d, ^3^*J* = 7 Hz), 144.04 (d, ^3^*J* = 3 Hz), 143.2 (d, ^2^*J* = 19 Hz), 140.1 (d, ^1^*J* = 246 Hz), 129.8 (d, ^3^*J* = 7 Hz), 124.1 (d, ^4^*J* = 3 Hz), 124.1 (d, ^4^*J* = 4 Hz), 115.3 (d, ^2^*J* = 20 Hz), 115.2 (d, ^2^*J* = 21 Hz), 113.8 (d, ^2^*J* = 22 Hz), 79.8, 72.1, 72.2, 48.4, 47.2, 44.0, 42.9, 38.7, 37.3, 31.5, 28.4, 19.8, 19.8, 14.0.

*(R)-tert-Butyl-4-(5-fluoro-4-((4-(4-fluorophenyl)butan-2-yl)oxy)pyrimidin-2-yl)-3-methylpiperazine-1-carboxylate* (**7i**): Yield: 40%; ^1^H-NMR (400 MHz, CDCl_3,_ Diastereomeric mixture) *δ* 7.96–7.95 (m, 1H, 1H′), 7.12–7.07 (m, 2H, 2H′), 6.96–6.91 (m, 2H, 2H′), 5.26–5.18 (m, 1H, 1H′), 4.64 (bs, 1H, 1H′), 4.22–3.89 (m, 3H, 3H′), 3.14–3.05 (m, 2H, 2H′), 2.89–2.62 (m, 3H, 3H′), 2.13–2.05 (m, 1H, 1H′) 1.94–1.85 (m, 1H, 1H′), 1.48 (d, *J* = 0.6 Hz, 9H, 9H′), 1.38 (d, *J* = 6.2 Hz, 3H, 3H′), 1.13–1.11 (m, 3H, 3H′). ^13^C-NMR (100 MHz, CDCl_3_, Diastereomeric mixture) *δ* 161.3 (d, ^1^*J* = 242 Hz), 157.9 (d, ^2^*J* = 10 Hz), 156.9, 155.2, 143.1 (d, ^2^*J* = 20 Hz), 140.2 (d, ^1^*J* = 246 Hz), 137.07 (d, ^4^*J* = 3 Hz), 137.06 (d, ^4^*J* = 2 Hz), 129.76 (d, ^3^*J* = 7 Hz), 129.72 (d, ^3^*J* = 7 Hz), 115.14 (d, ^2^*J* = 21 Hz), 115.12 (d, ^2^*J* = 21 Hz), 79.8, 72.20, 72.17, 48.5, 47.2, 44.0, 42.9, 38.7, 37.7, 30.99, 30.96, 28.4, 19.9, 19.8, 13.9.

#### 3.2.6. General Procedure for Preparing Compounds **4a**–**i**

Methods A: To a solution of methyl piperazine carboxylate **7** (0.161 mmol) in CH_2_Cl_2_ (1.60 mL), trifluoro acetic acid (4.01 mmol) was added at 0 °C. The reaction mixture was allowed to stir at the same temperature for 1 h. After completion of the reaction (monitored by TLC), the mixture was diluted with saturated aqueous NaHCO_3_, and extracted with EtOAc. The organic layers were dried over anhydrous MgSO_4_ and concentrated in vacuo. The resulting residue was purified by flash column chromatography on silica gel (DCM/MeOH = 10:1) to afford methyl piperazine **4**.

Methods B: To a solution of methyl piperazine carboxylate **7** (0.144 mmol) in dioxane (1.45 mL), 1 M HCl in ether (1.44 mmol) was added at 0 °C. The reaction mixture was allowed to stir at the same temperature for 2.5 h. After completion of the reaction (monitored by TLC), the mixture was diluted with saturated aqueous NaHCO_3_, and extracted with EtOAc. The organic layers were dried over anhydrous MgSO_4_ and concentrated in vacuo. The resulting residue was purified by flash column chromatography on silica gel (DCM/MeOH = 10:1) to afford methyl piperazine **4**.

*(R)-5-Fluoro-4-((2-fluorobenzyl)oxy)-2-(2-methylpiperazin-1-yl)pyrimidine* (**4a**): Methods A: Yield: 52%; ^1^H-NMR (400 MHz, MeOD) *δ* 8.16 (d, *J* = 3.1 Hz, 1H), 7.67–7.63 (m, 1H), 7.56–7.50 (m, 1H), 7.37–7.27 (m, 2H), 5.71–5.61 (m, 2H), 4.86–4.83 (m, 1H), 4.49–4.45 (m, 1H), 3.24–3.17 (m, 2H), 3.09–3.01 (m, 2H), 2.87–2.80 (m, 1H), 1.35 (d, *J* = 6.8 Hz, 3H). ^13^C-NMR (100 MHz, MeOD) *δ* 160.9 (d, ^1^*J* = 245 Hz), 157.6 (d, ^2^*J* = 11 Hz), 156.9 (d, ^4^*J* = 2 Hz), 143.0 (d, ^2^*J* = 20 Hz), 139.6 (d, ^1^*J* = 246 Hz), 130.1 (d, ^4^*J* = 3 Hz), 130.0 (d, ^3^*J* = 8 Hz), 124.1 (d, ^4^*J* = 3 Hz), 123.4 (d, ^2^*J* = 14 Hz), 115.0 (d, ^2^*J* = 21 Hz), 61.5 (d, ^3^*J* = 5 Hz), 49.1, 46.3, 44.9, 38.8, 12.4; HRMS-ESI (*m*/*z*): [M + H]^+^ calcd. for C_16_H_19_F_2_N_4_O: 321.1521 ; found: 321.1525; HPLC purity, 6.7 min, 98.8%.

*(R)-5-Fluoro-4-((3-fluorobenzyl)oxy)-2-(2-methylpiperazin-1-yl)pyrimidine* (**4b**): Methods A: Yield: 72%; ^1^H-NMR (400 MHz, MeOD) *δ* 8.18 (d, *J* = 2.9 Hz, 1H), 7.67–7.54 (m, 1H), 7.43 (d, *J* = 7.6 Hz, 1H), 7.36 (d, *J* = 9.6 Hz, 1H), 7.31–7.21 (m, 1H), 5.66–5.58 (m, 2H), 4.83 (bs, 1H), 4.47 (d, *J* =12.6 Hz, 1H), 3.25–3.18 (m, 2H), 3.10–3.02 (m, 2H), 2.88–2.81 (m, 1H), 1.35 (d, *J* = 6.8 Hz, 3H). ^13^C-NMR (100 MHz, MeOD) *δ* 162.9 (d, ^1^*J* = 243 Hz), 157.6 (d, ^2^*J* = 11 Hz), 156.9 (d, ^4^*J* = 2 Hz), 143.1 (d, ^2^*J* = 20 Hz), 139.6 (d, ^1^*J* = 245 Hz), 139.3 (d, ^3^*J* = 8 Hz), 130.0 (d, ^3^*J* = 8 Hz), 123.0 (d, ^4^*J* = 3 Hz), 114.4 (d, ^2^*J* = 21 Hz), 114.0 (d, ^2^*J* = 22 Hz), 66.9 (d, ^4^*J* = 1 Hz), 49.2, 46.4, 44.8, 38.8, 12.4; HRMS-ESI (*m/z*): [M + H]^+^ calcd. for C_16_H_19_F_2_N_4_O: 321.1521; found: 321.1523; HPLC purity, 6.9 min, 97.1%.

*(R)-5-Fluoro-4-((4-fluorobenzyl)oxy)-2-(2-methylpiperazin-1-yl)pyrimidine* (**4c**): Methods A: Yield: 57%; ^1^H-NMR (400 MHz, MeOD) *δ* 8.19 (d, *J* = 3.1 Hz, 1H), 7.68–7.65 (m, 2H), 7.30 (t, *J* = 8.8 Hz, 2H), 5.65–5.57 (m, 2H), 4.89–4.86 (m, 1H), 4.53–4.48 (m, 1H), 3.28–3.21 (m, 2H), 3.14–3.06 (m, 2H), 2.92–2.84 (m, 1H), 1.38 (d, *J* = 6.9 Hz, 3H). ^13^C-NMR (100 MHz, MeOD) *δ* 162.6 (d, ^1^*J* = 243 Hz), 157.7 (d, ^2^*J* = 11 Hz), 156.9 (d, ^4^*J* = 2 Hz), 143.0 (d, ^2^*J* = 20 Hz), 139.7 (d, ^1^*J* = 246 Hz), 132.5 (d, ^4^*J* = 4 Hz), 129.8 (d, ^3^*J* = 9 Hz), 114.9 (d, ^2^*J* = 22 Hz), 67.0, 49.1, 46.3, 44.8, 38.7, 12.4; HRMS-ESI (*m/z*): [M + H]^+^ calcd. for C_16_H_19_F_2_N_4_O: 321.1521; found: 321.1524; HPLC purity, 7.4 min, 97.4%.

*5-Fluoro-4-(1-(3-fluorophenyl)ethoxy)-2-((R)-2-methylpiperazin-1-yl)pyrimidine* (**4d**): Methods A: Yield: 71%; ^1^H-NMR (400 MHz, MeOD, Diastereomeric mixture) *δ* 8.13 (s, 1H, 1H′), 7.52–7.49 (m, 1H, 1H′), 7.39–7.36 (m, 1H, 1H′), 7.32–7.29 (m, 1H, 1H′), 7.16–7.14 (m, 2H), 6.31–6.22 (m, 1H, 1H′), 4.71 (bs, 1H, 1H′), 4.43–4.33 (m, 1H, 1H′), 3.18–2.99 (m, 4H, 4H′), 2.86–2.72 (m, 1H, 1H′), 1.83–1.81 (m, 3H, 3H′), 1.39–1.12 (m, 3H, 3H′). ^13^C-NMR (100 MHz, MeOD, Diastereomeric mixture) *δ* 162.93 (d, ^1^*J* = 245 Hz), 162.91 (d, ^1^*J* = 245 Hz), 157.13 (d, ^2^*J* = 11 Hz), 157.10 (d, ^2^*J* = 11 Hz), 156.8, 145.6 (d, ^3^*J* = 7 Hz), 145.3 (d, ^3^*J* = 7 Hz), 143.1 (d, ^2^*J* = 20 Hz), 143.0 (d, ^2^*J* = 19 Hz), 139.7 (d, ^1^*J* = 246 Hz), 130.1 (d, ^3^*J* = 8 Hz), 130.0 (d, ^3^*J* = 8 Hz), 121.14 (d, ^2^*J* = 27 Hz), 121.11 (d, ^2^*J* = 27 Hz), 114.0 (d, ^2^*J* = 22 Hz), 113.9 (d, ^2^*J* = 21 Hz), 74.05 (d, ^4^*J* = 2 Hz), 73.97 (d, ^4^*J* = 1 Hz), 49.1, 49.0, 46.3, 44.8, 44.7, 38.72, 38.68, 22.0, 21.8, 12.5, 12.4; HRMS-ESI (*m/z*): [M + H]^+^ calcd. for C_17_H_21_F_2_N_4_O: 335.1678 ; found: 335.1680; HPLC purity, 7.5 min, 98.1%.

*5-Fluoro-4-(1-(4-fluorophenyl)ethoxy)-2-((R)-2-methylpiperazin-1-yl)pyrimidine* (**4e**): Methods B: Yield: 53%; ^1^H-NMR (400 MHz, MeOD, Diastereomeric mixture) *δ* 8.16–8.14 (m, 1H, 1H′), 7.64–7.59 (m, 2H, 2H′), 7.28–7.23 (m, 2H, 2H′), 6.33–6.28 (m, 1H, 1H′), 4.78–4.76 (m, 1H, 1H′), 4.45–4.36 (m, 1H, 1H′), 3.21–3.10 (m, 2H, 2H′), 3.08–2.99 (m, 2H, 2H′), 2.89–2.75 (m, 1H, 1H′), 1.84–1.82 (m, 3H, 3H′), 1.41–1.17 (m, 3H, 3H′). ^13^C-NMR (100 MHz, MeOD, Diastereomeric mixture) *δ* 162.3 (d, ^1^*J* = 243 Hz), 162.2 (d, ^1^*J* = 243 Hz), 157.27 (d, ^2^*J* = 11 Hz), 157.25 (d, ^2^*J* = 11 Hz), 156.8, 142.93 (d, ^2^*J* = 20 Hz), 142.87 (d, ^2^*J* = 20 Hz), 139.81 (d, ^1^*J* = 243 Hz), 139.78 (d, ^1^*J* = 244 Hz), 138.4 (d, ^4^*J* = 3 Hz), 127.5 (d, ^3^*J* = 8 Hz), 127.3 (d, ^3^*J* = 8 Hz), 114.9 (d, ^2^*J* = 22 Hz), 114.8 (d, ^2^*J* = 22 Hz), 74.1, 74.0, 48.98, 48.94, 46.2, 44.71, 44.68, 38.6, 38.5, 22.0, 21.8, 12.4; HRMS-ESI (*m/z*): [M + H]^+^ calcd. for C_17_H_21_F_2_N_4_O: 335.1678 ; found: 335.1680; HPLC purity, 7.4 min, 98.3%.

*(R)-5-Fluoro-4-(3-(3-fluorophenyl)propoxy)-2-(2-methylpiperazin-1-yl)pyrimidine* (**4f**): Methods A: Yield: 62%; ^1^H-NMR (400 MHz, CDCl_3_) *δ* 7.99 (d, *J* = 3.0 Hz, 1H), 7.30–7.24 (m, 1H), 7.00 (d, *J* = 7.6 Hz, 1H), 6.95–6.89 (m, 2H), 4.67–4.61 (m, 1H), 4.42–4.36 (m, 2H), 4.29–4.25 (m, 1H), 3.09–2.98 (m, 3H), 2.90 (d, *J* = 12.2 Hz, 1H), 2.83–2.77 (m, 3H), 2.18–2.11 (m, 2H), 1.24 (d, *J* = 6.8 Hz, 3H). ^13^C-NMR (100 MHz, MeOD) *δ* 163.0 (d, ^1^*J* = 243 Hz), 158.3 (d, ^2^*J* = 11 Hz), 156.2, 144.1 (d, ^3^*J* = 8 Hz), 142.9 (d, ^2^*J* = 21 Hz), 140.3 (d, ^1^*J* = 247 Hz), 129.7 (d, ^3^*J* = 8 Hz), 124.1 (d, ^3^*J* = 4 Hz), 114.8 (d, ^2^*J* =21 Hz), 112.3 (d, ^2^*J* = 21 Hz), 65.5, 44.6, 43.3, 35.8, 31.3, 29.7, 12.3; HRMS-ESI (*m/z*): [M + H]^+^ calcd. for C_18_H_23_F_2_N_4_O: 349.1834; found: 347.1837; HPLC purity, 5.1 min, 98.7%.

*(R)-5-Fluoro-4-(3-(4-fluorophenyl)propoxy)-2-(2-methylpiperazin-1-yl)pyrimidine* (**4g**): Methods A: Yield: 63%; ^1^H-NMR (400 MHz, CDCl_3_) *δ* 7.93 (d, *J* = 3.0 Hz, 1H), 7.14–7.11 (m, 2H), 6.96–6.92 (m, 2H), 4.58 (t, *J* = 5.0 Hz, 1H), 4.35–4.31 (m, 2H), 4.23–4.19 (m, 1H), 3.03–2.92 (m, 3H), 2.84 (d, *J* = 12.3 Hz, 1H), 2.75–2.71 (m, 3H), 2.10–2.03 (m, 2H), 1.18 (d, *J* = 6.8 Hz, 3H). ^13^C-NMR (100 MHz, MeOD) *δ* 161.4 (d, ^1^*J* = 241 Hz), 158.4 (d, ^2^*J* = 11 Hz), 156.0, 142.8 (d, ^2^*J* = 20 Hz), 140.5 (d, ^1^*J* = 247 Hz), 137.1, 129.8 (d, ^3^*J* = 8 Hz), 114.6 (d, ^2^*J* = 21 Hz), 65.7, 46.9, 44.3, 43.1, 35.3, 30.7, 30.1, 12.4; HRMS-ESI (*m/z*): [M + H]^+^ calcd. for C_18_H_23_F_2_N_4_O: 349.1834; found: 347.1836; HPLC purity, 5.2 min, 95.5%.

*5-Fluoro-4-((4-(3-fluorophenyl)butan-2-yl)oxy)-2-((R)-2-methylpiperazin-1-l)pyrimidine* (**4h**): Methods A: Yield: 68%; ^1^H-NMR (400 MHz, MeOD, Diastereomeric mixture) δ 8.16–8.15 (m, 1H, 1H′), 7.46–7.40 (m, 1H, 1H′), 7.16–7.15 (m, 1H, 1H′), 7.09–7.05 (m, 2H, 2H′), 5.48–5.38 (m, 1H, 1H′), 4.80–4.72 (m, 1H, 1H′), 4.42–4.37 (m, 1H, 1H′), 3.24–3.15 (m, 2H, 2H′), 3.12–3.03 (m, 1H, 1H′), 2.99–2.83 (m, 3H, 3H′), 2.31–2.24 (m, 1H, 1H′), 2.18–2.09 (m, 1H, 1H′), 1.57–1.56 (m, 3H, 3H′), 1.39–1.35 (m, 3H, 3H′). ^13^C-NMR (100 MHz, MeOD, Diastereomeric mixture) *δ* 162.9 (d, ^1^*J* = 242 Hz), 157.86 (d, ^2^*J* = 11 Hz), 157.84 (d, ^2^*J* = 11 Hz), 156.9, 144.3 (d, ^3^*J* = 7 Hz), 142.7 (d, ^2^*J* = 20 Hz), 139.8 (d, ^1^*J* = 245 Hz), 129.68 (d, ^3^*J* = 9 Hz), 129.66 (d, ^3^*J* = 9 Hz), 124.02 (d, ^4^*J* = 3 Hz), 123.98 (d, ^4^*J* = 3 Hz), 114.8 (d, ^2^*J* = 21 Hz), 114.7 (d, ^2^*J* = 21 Hz), 112.3 (d, ^2^*J* = 22 Hz), 112.2 (d, ^2^*J* = 22 Hz), 72.01, 71.92, 49.12, 49.10, 46.24, 46.22, 44.82, 44.80, 38.70, 38.68, 36.9, 31.0, 18.66, 18.64, 12.4, 12.3; HRMS-ESI (*m/z*): [M + H]^+^ calcd. for C_19_H_25_F_2_N_4_O: 363.1991; found: 363.1992; HPLC purity, 8.0 min, 98.4%.

*5-Fluoro-4-((4-(4-fluorophenyl)butan-2-yl)oxy)-2-((R)-2-methylpiperazin-1-yl)pyrimidine* (**4i**): Methods A: Yield: 53%; ^1^H-NMR (400 MHz, MeOD, Diastereomeric mixture) *δ* 8.12 (d, *J* = 2.4 Hz, 1H, 1H′), 7.32–7.29 (m, 2H, 2H′), 7.14–7.08 (m, 2H, 2H′), 5.43–5.34 (m, 1H, 1H′), 4.74–4.67 (m, 1H, 1H′), 4.35–4.31 (m, 1H, 1H′), 3.18–3.11 (m, 2H, 2H′), 3.08–2.99 (m, 2H, 2H′), 2.93–2.79 (m, 1H, H′), 2.28–2.19 (m, 1H, 1H′) 2.13–2.03 (m, 1H, 1H′), 1.54–1.51 (m, 3H, 3H′), 1.35–1.31 (m, 3H, 3H′). ^13^C-NMR (100 MHz, MeOD, Diastereomeric mixture) *δ* 161.3 (d, ^1^*J* = 241 Hz), 157.87 (d, ^2^*J* = 11 Hz), 157.85 (d, ^2^*J* = 11 Hz), 156.9, 142.7 (d, ^2^*J* = 20 Hz), 139.9 (d, ^1^*J* = 246 Hz), 137.3 (d, ^4^*J* = 4 Hz), 129.72 (d, ^3^*J* = 7 Hz), 129.67 (d, ^3^*J* = 7 Hz), 114.57 (d, ^2^*J* = 21 Hz), 114.55 (d, ^2^*J* = 21 Hz), 72.00, 71.95, 49.13, 49.09, 46.24, 46.22, 44.83, 44.79, 38.71, 38.68, 37.3, 30.5, 30.4, 18.66, 18.62, 12.4, 12.3; HRMS-ESI (*m/z*): [M + H]^+^ calcd. for C_19_H_25_F_2_N_4_O: 363.1991; found: 363.1993; HPLC purity, 8.2 min, 96.9%.

### 3.3. Synthesis of Optically Active Pyrimidines ***4d*** and ***4e***

#### 3.3.1. General Procedure for Preparing Compound (*R*)-(+)-**5d** and (*R*)-(+)-**5e**

To a solution of 1-(3 or 4-fluorophenyl)ethanol (6.67 mmol) in *n*-hexane (22.2 mL), CAL-B (147 mg), vinyl acetate (3.34 mmol), and triethylamine (0.667 mmol) were added. The reaction mixture was allowed to stir at room temperature for 1 h. After completion of the reaction (monitored by TLC), the mixture was filtered and concentrated in vacuo. The resulting residue was purified by flash column chromatography on silica gel (EtOAc:*n*-hexane = 1:8) to afford acetate intermediate (315 mg) as a colorless oil. To a solution of acetate (1.73 mmol) in MeOH (3.45 mL), 1 M NaOH (2.59 mmol) was added. The reaction mixture was allowed to stir at room temperature for 1 h. After completion of the reaction (monitored by TLC), it was quenched with ditilled water and extracted with EtOAc. The organic layers were dried over anhydrous MgSO_4_ and concentrated in vacuo. The resulting residue was purified by flash column chromatography on silica gel (EtOAc:*n*-hexane = 1:8) to afford alcohol (*R*)-(+)-**5d** and (*R*)-(+)-**5e**.

*(R)-1-(3-Fluorophenyl)ethan-1-ol* ((*R*)-(+)**-5d**): Yield: 17%; ^1^H-NMR (400 MHz, CDCl_3_) *δ* 7.32–7.26 (m, 1H), 7.12–7.07 (m, 2H), 6.97–6.92 (m, 1H), 4.87 (q, *J* = 6.4 Hz, 1H), 2.18 (s, 1H), 1.47 (d, *J* = 6.4 Hz, 3H). ^13^C-NMR (100 MHz, CDCl_3_) *δ* 163.0 (d, ^1^*J* = 244 Hz), 148.5 (d, ^3^*J* = 6 Hz), 130.0 (d, ^3^*J* = 8 Hz), 121.0 (d, ^4^*J* = 3 Hz), 114.2 (d, ^2^*J* = 21 Hz), 112.3 (d, ^2^*J* = 21 Hz), 69.8, 25.2; Optical rotation for (*R*)-(+)-**5d**: ${[\alpha ]\;}_{D}^{26}$ +43.7° (*c* 0.7, CHCl_3_).

*(R)-1-(4-Fluorophenyl)ethan-1-ol* ((*R*)-(+)**-5e**): Yield: 32%; ^1^H-NMR (400 MHz, CDCl_3_) *δ* 7.32–7.26 (m, 2H), 7.03–6.97 (m, 2H), 4.84 (q, *J* = 6.1 Hz, 1H), 2.34 (s, 1H), 1.44 (d, *J* = 6.4 Hz, 3H). ^13^C-NMR (100 MHz, CDCl_3_) *δ* 162.1 (d, ^1^*J* = 244 Hz), 141.6 (d, ^4^*J* = 3 Hz), 127.1 (d, ^3^*J* = 8 Hz), 115.2 (d, ^2^*J* = 21 Hz), 69.7, 25.3; Optical rotation for (*R*)-(+)-**5e**: ${\left[\alpha \right]}_{D}^{27}$ +51.9° (*c* 0.5, CHCl_3_).

#### 3.3.2. General Procedure for Preparing Compound (*S*)-(−)-**5d** and (*S*)-(−)-**5e**

To a solution of 1-(3 or 4-fluorophenyl)ethanol (6.67 mmol) in *n*-hexane (22.2 mL), CAL-B (147 mg), vinyl acetate (13.3 mmol), and triethylamine (0.667 mmol) were added. The reaction mixture was allowed to stir at room temperature for 12 h. After completion of the reaction (monitored by TLC), the mixture was filtered and concentrated in vacuo. The resulting residue was purified by flash column chromatography on silica gel (EtOAc:*n*-hexane = 1:8) to afford alcohol (*S*)-(−)-**5d** and (*S*)-(−)-**5e**.

*(S)-1-(3-Fluorophenyl)ethan-1-ol* ((*S*)-(−)**-5d**): Yield: 44%; ^1^H-NMR (400 MHz, CDCl_3_) *δ* 7.31–7.26 (m, 1H), 7.11–7.06 (m, 2H), 6.96–6.91 (m, 1H), 4.85 (td, *J* = 5.5, 7.5 Hz, 1H), 2.31 (s, 1H), 1.46 (d, *J* = 6.5 Hz, 3H). ^13^C-NMR (100 MHz, CDCl_3_) *δ* 163.0 (d, ^1^*J* = 245 Hz), 148.6 (d, ^3^*J* = 6 Hz), 130.0 (d, ^3^*J* = 8 Hz), 121.0 (d, ^4^*J* = 3 Hz), 114.2 (d, ^2^*J* = 21 Hz), 112.3 (d, ^2^*J* = 22 Hz), 69.8 (d, ^4^*J* = 2 Hz), 25.2; Optical rotation for (*S*)-(−)**-5d**: ${[\alpha ]\;}_{D}^{27}$ −46.9° (*c* 0.4, CHCl_3_).

*(S)-1-(4-Fluorophenyl)ethan-1-ol* ((*S*)-(−)**-5e**): Yield: 46%; ^1^H-NMR (400 MHz, CDCl_3_) *δ* 7.33–7.30 (m, 2H), 7.04–6.98 (m, 2H), 4.85 (q, *J* = 6.4 Hz, 1H), 2.16 (s, 1H), 1.45 (d, *J* = 6.4 Hz, 3H). ^13^C-NMR (100 MHz, CDCl_3_) *δ* 162.1 (d, ^1^*J* = 243 Hz), 141.5 (d, ^4^*J* = 3 Hz), 127.1 (d, ^3^*J* = 8 Hz), 115.2 (d, ^2^*J* = 21 Hz), 69.8, 25.3; Optical rotation for (*S*)-(−)**-5e**: ${[\alpha ]\;}_{D}^{27}$ –49.7° (*c* 0.6, CHCl_3_).

#### 3.3.3. General Procedure for Preparing Compounds (*R*)/(*S*)-**10** and **11**

To a solution of (*R*) or (*S*)-secondary alcohol (0.0749 mmol) in CH_2_Cl_2_ (0.400 mL), (*R*)-2-acetoxy-2-phenylacetic acid (0.112 mmol), EDCI (0.112 mmol), and DMAP (0.112 mmol) were added. The reaction mixture was allowed to stir at room temperature for 12 h. After completion of the reaction (monitored by TLC), the mixture was filtered, extracted with CH_2_Cl_2_, and washed with brine. The organic layers were dried over anhydrous MgSO_4_ and concentrated in vacuo. The resulting residue was purified by flash column chromatography on silica gel (EtOAc:*n*-hexane = 1:8) to afford mandelate (*R*)/(*S*)-**10** and **11**.

*(R)-1-(3-Fluorophenyl)ethyl (R)-2-acetoxy-2-phenylacetate* ((*R*)**-10**): Yield: 86%; ^1^H-NMR (400 MHz, CDCl_3_) *δ* 7.41–7.34 (m, *3*H), 7.18–7.12 (m, 1H), 6.91–6.86 (m, 1H), 6.79 (d, *J* = 7.7 Hz, 1H), 6.67 (dd, *J* = 1.9, 9.8 Hz, 1H), 5.97 (s, 1H), 5.85 (q, *J* = 6.6 Hz, 1H), 2.19 (s, 3H), 1.52 (d, *J* = 6.6 Hz, 3H).

*(S)-1-(3-Fluorophenyl)ethyl (R)-2-acetoxy-2-phenylacetate* ((*S*)**-10**): Yield: 84%; ^1^H-NMR (400 MHz, CDCl_3_) *δ* 7.50–7.47 (m, 2H), 7.44–7.40 (m, 3H), 7.33–7.26 (m, 1H), 7.09 (d, *J* = 7.7 Hz, 1H ), 7.04–6.96 (m, 2H), 5.96 (s, 1H), 5.88 (q, *J* = 6.6 Hz, 1H), 2.19 (s, 3H), 1.41 (d, *J* = 6.6 Hz, 3H).

*(R)-1-(4-Fluorophenyl)ethyl (R)-2-acetoxy-2-phenylacetate* ((*R*)**-11**): Yield: 68%; ^1^H-NMR (400 MHz, CDCl_3_) *δ* 7.39–7.31 (m, 5H), 6.99–6.95 (m, 2H), 6.89–6.83 (m, 2H), 5.94 (s, 1H), 5.84 (q, *J* = 6.6 Hz, 1H), 2.17 (s, 3H), 1.52 (d, *J* = 6.6 Hz, 3H).

*(S)-1-(4-Fluorophenyl)ethyl (R)-2-acetoxy-2-phenylacetate* ((*S*)**-11**): Yield: 84%; ^1^H-NMR (400 MHz, CDCl_3_) *δ* 7.48–7.45 (m, 2H), 7.41–7.37 (m, 3H), 7.31–7.25 (m, 2H), 7.05–7.00 (m, 2H), 5.93 (s, 1H), 5.87 (q, *J* = 6.6 Hz, 1H), 2.18 (s, 3H), 1.40 (d, *J* = 6.6 Hz, 3H).

#### 3.3.4. General Procedure for Preparing Compounds (*R*,*R*)- or (*S*,*R*)-**4d**, and **4e**

The title compounds (*R,R*) or (*S,R*)-**4d** and **4**e were prepared from (*R*)-(+)-**5d/5e** and (*S*)-(+)-**5d/5e** following the same procedures described for the synthesis of **4d** and **4e**.

*(R)-2-Chloro-5-fluoro-4-(1-(3-fluorophenyl)ethoxy)pyrimidine* ((*R*)**-6d**): Yield: 72%; ^1^H-NMR (400 MHz, CDCl_3_) *δ* 8.19 (d, *J* = 2.2 Hz, 1H), 7.37–7.31 (m, 1H), 7.23 (d, *J* = 7.7 Hz, 1H), 7.18–7.15 (m, 1H), 7.03–6.99 (m, 1H), 6.30 (q, *J* = 6.5 Hz, 1H), 1.71 (d, *J* = 6.6 Hz, 3H). ^13^C-NMR (100 MHz, CDCl_3_) *δ* 162.9 (d, ^1^*J* = 245 Hz), 158.6 (d, ^2^*J* = 11 Hz), 153.2 (d, ^4^*J* = 5 Hz), 146.0 (d, ^1^*J* = 263 Hz), 144.4 (d, ^2^*J* = 20 Hz), 142.9 (d, ^3^*J* = 7 Hz), 130.3 (d, ^3^*J* = 8 Hz), 122.0 (d, ^4^*J* = 3 Hz), 115.3 (d, ^2^*J* = 21 Hz), 113.3 (d, ^2^*J* = 22 Hz), 75.6 (d, ^4^*J* = 2 Hz), 22.2; Optical rotation for (*R*)-**6d**: ${[\alpha ]\;}_{D}^{27}$ +178.3° (*c* 0.7, CHCl_3_).

*(S)-2-Chloro-5-fluoro-4-(1-(3-fluorophenyl)ethoxy)pyrimidine* ((*S*)**-6d**): Yield: 55%; ^1^H-NMR (400 MHz, CDCl_3_) *δ* 8.18 (d, *J* = 2.2 Hz, 1H), 7.36–7.31 (m, 1H), 7.22 (d, *J* = 7.7 Hz, 1H), 7.18–7.14 (m, 1H), 7.03–6.98 (m, 1H), 6.30 (q, *J* = 6.5 Hz, 1H), 1.71 (d, *J* = 6.6 Hz, 3H). ^13^C-NMR (100 MHz, CDCl_3_) *δ* 162.9 (d, ^1^*J* = 245 Hz), 158.6 (d, ^2^*J* = 11 Hz), 153.2 (d, ^4^*J* = 4 Hz), 146.0 (d, ^1^*J* = 262 Hz), 144.4 (d, ^2^*J* = 20 Hz), 142.9 (d, ^3^*J* = 7 Hz), 130.3 (d, ^3^*J* = 8 Hz), 122.0 (d, ^4^*J* = 3 Hz), 115.3 (d, ^2^*J* = 21 Hz), 113.3 (d, ^2^*J* = 22 Hz), 75.7 (d, ^4^*J* = 1 Hz), 22.2; Optical rotation for (*S*)-**6d**: ${[\alpha ]\;}_{D}^{28}$ −182.7° (*c* 0.7, CHCl_3_).

*(R)-2-Chloro-5-fluoro-4-(1-(4-fluorophenyl)ethoxy)pyrimidine* ((*R*)**-6e**): Yield: 68%; ^1^H-NMR (400 MHz, CDCl_3_) *δ* 8.16 (d, *J* = 2.2 Hz, 1H), 7.47–7.42 (m, 2H), 7.08–7.02 (m, 2H), 6.30 (q, *J* = 6.6 Hz, 1H), 1.71 (d, *J* = 6.6 Hz, 3H). ^13^C-NMR (100 MHz, CDCl_3_) *δ* 162.6 (d, ^1^*J* = 245 Hz), 158.7 (d, ^2^*J* = 11 Hz), 153.1 (d, ^4^*J* = 4 Hz), 146.0 (d, ^1^*J* = 263 Hz), 144.3 (d, ^2^*J* = 20 Hz), 136.1 (d, ^4^*J* = 4 Hz), 128.4 (d, ^3^*J* = 9 Hz), 115.6 (d, ^2^*J* = 22 Hz), 75.9, 22.2; Optical rotation for (*R*)-**6e**: ${[\alpha ]\;}_{D}^{27}$ +197.3° (*c* 0.8, CHCl_3_).

*(S)-2-Chloro-5-fluoro-4-(1-(4-fluorophenyl)ethoxy)pyrimidine* ((*S*)**-6e**): Yield: 65%; ^1^H-NMR (400 MHz, CDCl_3_) *δ* 8.16 (d, *J* = 2.2 Hz, 1H), 7.47–7.42 (m, 2H), 7.08–7.02 (m, 2H), 6.30 (q, *J* = 6.6 Hz, 1H), 1.71 (d, *J* = 6.6 Hz, 3H). ^13^C-NMR (100 MHz, CDCl_3_) *δ* 162.6 (d, ^1^*J* = 245 Hz), 158.7 (d, ^2^*J* = 11 Hz), 153.1 (d, ^4^*J* = 4 Hz), 146.0 (d, ^1^*J* = 263 Hz), 144.3 (d, ^2^*J* = 20 Hz), 136.1 (d, ^4^*J* = 3 Hz), 128.4 (d, ^3^*J* = 8 Hz), 115.7 (d, ^2^*J* = 21 Hz), 75.9, 22.2; Optical rotation for (*S*)-**6e**: ${[\alpha ]\;}_{D}^{27}$ −204.7° (*c* 0.8, CHCl_3_).

*tert-Butyl-(R)-4-(5-fluoro-4-((R)-1-(3-fluorophenyl)ethoxy)pyrimidin-2-yl)-3-methylpiperazine-1-carboxylate* ((*R,R*)**-7d**): Yield: 50%; ^1^H-NMR (400 MHz, CDCl_3_) *δ* 7.94 (d, *J* = 2.8 Hz, 1H), 7.31–7.26 (m, 1H), 7.14 (d, *J* = 7.7 Hz, 1H), 7.07 (d, *J* = 9.6 Hz, 1H) 6.96–6.91 (m, 1H), 6.03 (q, *J* = 6.6 Hz, 1H), 4.58 (bs, 1H), 4.18–3.86 (m, 3H), 3.08–3.01 (m, 2H), 2.84–2.74 (m, 1H), 1.66 (d, *J* = 6.6 Hz, 3H), 1.47 (s, 9H), 0.91 (d, *J* = 6.5 Hz, 3H). ^13^C-NMR (100 MHz, CDCl_3_) *δ* 162.9 (d, ^1^*J* = 245 Hz), 157.1 (d, ^2^*J* = 11 Hz), 156.7 (d, ^4^*J* = 2 Hz), 155.2, 145.1 (d, ^3^*J* = 7 Hz), 143.4 (d, ^2^*J* = 20 Hz), 140.0 (d, ^1^*J* = 246 Hz), 130.1 (d, ^3^*J* = 8 Hz), 121.2 (d, ^4^*J* = 3 Hz), 114.5 (d, ^2^*J* = 21 Hz), 112.7 (d, ^2^*J* = 22 Hz), 79.8, 73.9, 48.4, 47.1, 43.9, 42.8, 38.7, 28.4, 23.0, 13.9; Optical rotation for (*R,R*)-**7d**: ${[\alpha ]\;}_{D}^{27}$ +105.0° (*c* 0.4, CHCl_3_).

*tert-Butyl-(R)-4-(5-fluoro-4-((S)-1-(3-fluorophenyl)ethoxy)pyrimidin-2-yl)-3-methylpiperazine-1-carboxylate* ((*S,R*)**-7d**): Yield: 57%; ^1^H-NMR (400 MHz, CDCl_3_) *δ* 7.95 (d, *J* = 2.9 Hz, 1H), 7.32–7.26 (m, 1H), 7.16 (d, *J* = 7.7 Hz, 1H), 7.11–7.09 (m, 1H) 6.97–6.93 (m, 1H), 6.06 (q, *J* = 6.6 Hz, 1H), 4.55 (bs, 1H), 4.22–3.84 (m, 3H), 3.07–2.87 (m, 3H), 1.66 (d, *J* = 6.6 Hz, 3H), 1.47 (s, 9H), 1.13 (d, *J* = 6.6 Hz, 3H). ^13^C-NMR (100 MHz, CDCl_3_) *δ* 163.0 (d, ^1^*J* = 245 Hz), 157.1 (d, ^2^*J* = 11 Hz), 156.7 (d, ^4^*J* = 2 Hz), 155.2, 144.9 (d, ^3^*J* = 7 Hz), 143.5 (d, ^2^*J* = 19 Hz), 140.0 (d, ^1^*J* = 247 Hz), 130.0 (d, ^3^*J* = 8 Hz), 121.4 (d, ^4^*J* = 3 Hz), 114.6 (d, ^2^*J* = 21 Hz), 112.8 (d, ^2^*J* = 22 Hz), 79.8, 73.8, 48.4, 47.1, 43.9, 42.9, 38.7, 28.4, 22.8, 14.0; Optical rotation for (*S,R*)-**7d**: ${[\alpha ]\;}_{D}^{27}$ –215.3° (*c* 0.7, CHCl_3_).

*tert-Butyl-(R)-4-(5-fluoro-4-((R)-1-(4-fluorophenyl)ethoxy)pyrimidin-2-yl)-3-methylpiperazine-1-carboxylate* ((*R,R*)**-7e**): Yield: 44%; ^1^H-NMR (400 MHz, CDCl_3_) *δ* 7.93 (d, *J* = 2.8 Hz, 1H), 7.39–7.33 (m, 2H), 7.04–6.98 (m, 2H), 6.07 (d, *J* = 6.6 Hz, 1H), 4.60 (bs, 1H), 4.23–3.86 (m, 3H), 3.09–2.77 (m, 3H), 1.65 (d, *J* = 6.6 Hz, 3H) 1.46 (s, 9H), 0.95 (d, *J* = 6.6 Hz, 3H). ^13^C-NMR (100 MHz, CDCl_3_) *δ* 162.2 (d, ^1^*J* = 245 Hz), 157.2 (d, ^2^*J* = 11 Hz), 156.7 (d, ^4^*J* = 2 Hz), 155.2, 143.3 (d, ^2^*J* = 19 Hz), 140.0 (d, ^1^*J* = 247 Hz), 138.1 (d, ^4^*J* = 3 Hz), 127.4 (d, ^3^*J* = 8 Hz), 115.4 (d, ^2^*J* = 21 Hz), 79.8, 73.9, 73.9, 48.4, 47.1, 43.7, 42.8, 38.7, 28.4, 23.0, 14.0; Optical rotation for (*R,R*)-**7e**: ${\left[\alpha \right]}_{D}^{28}$ +77.9° (*c* 0.4, CHCl_3_).

*tert-Butyl-(R)-4-(5-fluoro-4-((S)-1-(4-fluorophenyl)ethoxy)pyrimidin-2-yl)-3-methylpiperazine-1-carboxylate* ((*S,R*)**-7e**): Yield: 52%; ^1^H-NMR (400 MHz, CDCl_3_) *δ* 7.93 (d, *J* = 2.8 Hz, 1H), 7.39–7.32 (m, 2H), 7.04–6.95 (m, 2H), 6.09 (d, *J* = 6.5 Hz, 1H), 4.59 (bs, 1H), 4.24–3.85 (m, 3H), 3.09–2.88 (m, 3H), 1.65 (d, *J* = 6.6 Hz, 3H) 1.47 (s, 9H), 1.13 (d, *J* = 6.4 Hz, 3H). ^13^C-NMR (100 MHz, CDCl_3_) *δ* 162.3 (d, ^1^*J* = 245 Hz), 157.2 (d, ^2^*J* = 11 Hz), 156.7 (d, ^4^*J* = 3 Hz), 155.2, 143.4 (d, ^2^*J* = 19 Hz), 140.0 (d, ^1^*J* = 247 Hz), 137.9 (d, ^4^*J* = 3 Hz), 127.7 (d, ^3^*J* = 8 Hz), 115.4 (d, ^2^*J* = 22 Hz), 79.9, 73.8, 48.4, 47.2, 43.7, 42.9, 38.7, 28.4, 22.8, 14.0; Optical rotation for (*S,R*)-**7e**: ${[\alpha ]\;}_{D}^{28}$ −212.4° (*c* 0.5, CHCl_3_).

*5-Fluoro-4-((R)-1-(3-fluorophenyl)ethoxy)-2-((R)-2-methylpiperazin-1-yl)pyrimidine* ((*R,R*)**-4d**): Methods A: Yield: 67%; ^1^H-NMR (400 MHz, CDCl_3_) *δ* 7.99 (d, *J* = 2.4 Hz, 1H), 7.32–7.26 (m, 1H), 7.13 (d, *J* = 7.6 Hz, 1H), 7.06 (d, *J* = 9.5 Hz, 1H), 6.97–6.93 (m, 1H), 5.99 (q, *J* = 6.5 Hz, 1H), 4.88 (bs, 1H), 4.47 (d, *J* = 13.7 Hz, 1H), 3.41–3.14 (m, 4H), 2.84 (bs, 1H), 1.68 (d, *J* = 6.6 Hz, 3H), 1.12 (d, *J* = 6.9 Hz, 3H). ^13^C-NMR (100 MHz, CDCl_3_) *δ* 163.0 (d, ^1^*J* = 245 Hz), 157.6 (d, ^2^*J* = 11 Hz), 145.2 (d, ^4^*J* = 2 Hz), 144.8 (d, ^3^*J* = 7 Hz), 143.1 (d, ^2^*J* = 20 Hz), 140.6 (d, ^1^*J* = 246 Hz), 130.3 (d, ^3^*J* = 8 Hz), 121.0 (d, ^4^*J* = 3 Hz), 114.7 (d, ^2^*J* = 21 Hz), 112.5 (d, ^2^*J* = 23 Hz), 74.7, 47.2, 44.4, 43.1, 35.5, 23.0, 13.2; HRMS-ESI (*m/z*): [M + H]^+^ calcd for C_17_H_21_F_2_N_4_O: 335.1678; found: 335.1681; HPLC purity, 9.4 min, 98.4%; Optical rotation for (*R,R*)-**4d**: ${\left[\alpha \right]}_{D}^{26}$ +118.2° (*c* 0.3, MeOH).

*5-Fluoro-4-((S)-1-(3-fluorophenyl)ethoxy)-2-((R)-2-methylpiperazin-1-yl)pyrimidine* ((*S,R*)**-4d**): Methods A: Yield: 71%; ^1^H-NMR (400 MHz, CDCl_3_) *δ* 7.99 (d, *J* = 2.4 Hz, 1H), 7.34–7.26 (m, 1H), 7.15 (d, *J* = 7.7 Hz, 1H), 7.09 (d, *J* = 9.6 Hz, 1H), 7.00–6.95 (m, 1H), 6.03 (q, *J* = 6.5 Hz, 1H), 4.88–4.85 (m, 1H), 4.53 (d, *J* = 13.6 Hz, 1H), 3.43 (d, *J* = 12.1 Hz, 1H), 3.34–2.97 (m, 4H), 1.68 (d, *J* = 6.6 Hz, 3H), 1.36 (d, *J* = 7.1 Hz, 3H). ^13^C-NMR (100 MHz, CDCl_3_) *δ* 163.0 (d, ^1^*J* = 245 Hz), 157.7 (d, ^2^*J* = 11 Hz), 155.5 (d, ^4^*J* = 3 Hz), 144.5 (d, ^3^*J* = 7 Hz), 143.1 (d, ^2^*J* = 21 Hz), 140.5 (d, ^1^*J* = 249 Hz), 130.2 (d, ^3^*J* = 8 Hz), 121.2 (d, ^4^*J* = 3 Hz), 114.8 (d, ^2^*J* = 21 Hz), 112.7 (d, ^2^*J* = 22 Hz), 74.6, 47.0, 44.4, 43.2, 35.7, 22.3, 13.5; HRMS-ESI (*m/z*): [M + H]^+^ calcd for C_17_H_21_F_2_N_4_O: 335.1678; found: 335.1682; HPLC purity, 7.3 min, 97.4%; Optical rotation for (*S,R*)-**4d**: ${\left[\alpha \right]}_{D}^{27}$ −228.6° (*c* 0.4, MeOH).

*5-Fluoro-4-((R)-1-(4-fluorophenyl)ethoxy)-2-((R)-2-methylpiperazin-1-yl)pyrimidine* ((*R,R*)**-4e**): Methods B: Yield: 82%; ^1^H-NMR (400 MHz, CDCl_3_) *δ* 7.94 (d, *J* = 2.8 Hz, 1H), 7.40–7.34 (m, 2H), 7.04–6.99 (m, 2H), 6.08 (q, *J* = 6.6 Hz, 1H), 4.60–4.55 (m, 1H), 4.18 (d, *J* = 3.5 Hz, 1H), 3.06–2.86 (m, 4H), 2.72–2.65 (m, 1H), 2.54 (bs, 1H), 1.65 (d, *J* = 6.6 Hz, 1H), 1.04 (d, *J* = 6.8 Hz, 1H). ^13^C-NMR (100 MHz, CDCl_3_) *δ* 162.2 (d, ^1^*J* = 244 Hz), 157.2 (d, ^2^*J* = 11 Hz), 156.9 (d, ^4^*J* = 2 Hz), 143.3 (d, ^2^*J* = 19 Hz), 139.9 (d, ^1^*J* = 246 Hz), 138.2 (d, ^4^*J* = 3 Hz), 127.5 (d, ^3^*J* = 8 Hz), 115.4 (d, ^2^*J* = 21 Hz), 73.8, 50.3, 16.6, 45.8, 39.5, 23.0, 13.6; HRMS-ESI (*m*/*z*): [M + H]^+^ calcd for C_17_H_21_F_2_N_4_O: 335.1678; found: 335.1680; HPLC purity, 7.5 min, 95.7%; Optical rotation for (*R,R*)-**4e**: ${\left[\alpha \right]}_{D}^{28}$ +132.0° (*c* 0.3, MeOH).

*5-Fluoro-4-((S)-1-(4-fluorophenyl)ethoxy)-2-((R)-2-methylpiperazin-1-yl)pyrimidine* ((*S,R*)**-4e**): Methods B: Yield: 81%; ^1^H-NMR (400 MHz, CDCl_3_) *δ* 7.95 (d, *J* = 2.9 Hz, 1H), 7.40–7.37 (m, 2H), 7.05–7.00 (m, 2H), 6.11 (q, *J* = 6.6 Hz, 1H), 4.61–4.57 (m, 1H), 4.27–4.23 (m, 1H), 3.09–2.86 (m, 4H), 2.78–2.71 (m, 1H), 2.59 (bs, 1H), 1.66 (d, *J* = 6.6 Hz, 1H), 1.23 (d, *J* = 6.8 Hz, 1H). ^13^C-NMR (100 MHz, CDCl_3_) *δ* 162.3 (d, ^1^*J* = 244 Hz), 157.2 (d, ^2^*J* = 11 Hz), 156.9 (d, ^4^*J* = 2 Hz), 143.4 (d, ^2^*J* = 20 Hz), 140.0 (d, ^1^*J* = 246 Hz), 137.9 (d, ^4^*J* = 3 Hz), 127.7 (d, ^3^*J* = 8 Hz), 115.4 (d, ^2^*J* = 21 Hz), 73.7, 50.2, 46.5, 45.8, 39.5, 22.7, 13.6; HRMS-ESI (*m*/*z*): [M + H]^+^ calcd for C_17_H_21_F_2_N_4_O: 335.1678; found: 335.1678; HPLC purity, 7.4 min, 97.7%; Optical rotation for (*S,R*)-**4e**: ${\left[\alpha \right]}_{D}^{28}$ –226.5° (c 0.5, MeOH).

### 3.4. Serotonin Receptor Binding Affinity Assays

Eleven dilutions (5 × assay concentration) of the test and reference compounds (Table S1) were prepared in standard binding buffer (50 mM tris(hydroxymethyl)-aminomethane-HCl (Tris-HCl), 10 mM MgCl_2_, 1 mM ethylenediaminetetraacetate (EDTA), pH 7.4) by serial dilution: 0.05 nM, 0.5 nM, 1.5 nM, 5 nM, 15 nM, 50 nM, 150 nM, 500 nM, 1.5 μM, 5 μM, and 50 μM. The radioligand (Table S3) was diluted to five times the assay concentration in standard binding buffer. Aliquots (50 mL) of the radioligand were dispensed into the wells of a 96-well plate containing 100 mL of standard binding buffer. Triplicate aliquots (50 mL) of the test and reference compound dilutions were then added. Finally, crude membrane fractions (50 mL) of cells (HEK293 or CHO) expressing human recombinant receptors were dispensed into each well. A total of 250 mL of the reaction mixtures was incubated at room temperature and shielded from light for 1.5 h, and was then harvested by rapid filtration onto Whatman GF/B glass fiber filters presoaked with 0.3% polyethyleneimine, by using a 96-well Brandel harvester (Gaithersburg, MD, USA).

Four rapid washes were performed with chilled standard binding buffer (500 mL) to decrease nonspecific binding. Filters were placed in 6 mL scintillation tubes and allowed to dry overnight. The next day, 4-mL of EcoScint scintillation cocktail (National Diagnostics) was added to each tube. The tubes were capped, labeled, and counted by liquid scintillation counting. The filter mats were dried, and the scintillant was melted onto the filters, then the radioactivity retained on the filters was counted in a Microbeta scintillation counter. The IC_50_ values were obtained by using the Prism 4.0 program (GraphPad Software, La Jolla, CA, USA) and were converted into *K*_i_ values. Each compound was tested at least in triplicate.

## 4. Conclusions

In summary, we have synthesized a series of pyrimidine derivatives **4a**–**i** and evaluated their binding affinities towards 5-HT_2C_ receptors. Our initial biological study indicated that 2-amino-4-alkoxypyrimidines **4b**, **4d**, and **4e**, possessing a short carbon chain between the phenyl group and pyrimidine, have excellent 5-HT_2C_ binding affinities, which are comparable to that of the reported pyrimidine analogue **3**. In order to improve the selectivity for other 5-HT_2_ receptor subtypes, the most potent compounds **4d** and **4e** were selected and their diastereomeric isomers were synthesized as optically pure forms. For this purpose, optically active secondary alcohols **5d** and **5e** were also prepared by an enzymatic kinetic resolution. (*R,R*)-**4d** and **4e** displayed excellent 5-HT_2C_ binding affinities with less selectivity towards 5-HT_2A_ and 5-HT_2B_, whereas (*S,R*)-**4d** and **4e** exhibited low potencies for 5-HT_2A_ and 5-HT_2B_ with a slight loss of the 5-HT_2C_ binding affinity. These results suggest that the pyrimidine analogue (*R*,*R*)-**4e** is a potential lead compound for identifying a 5-HT_2C_ selective modulator.

## Figures and Tables

**Table 1:** Binding affinities of pyrimidine analogues **4a**–**i** against 5-HT_2_ receptor subtypes.

Entry	Comp.	*n*	F	R	5-HT_2A_		5-HT_2B_		5-HT_2C_	
					%Binding	*K*_i_ (nM)	%Binding	*K*_i_ (nM)	%Binding	*K*_i_ (nM)
1	**4a**	0	2–F	H	93.1	80.0	96.9	56.0	97.8	31.0
2	**4b**	0	3–F	H	97.4	43.0	98.5	14.0	99.0	5.1
3	**4c**	0	4–F	H	94.6	51.0	97.6	14.0	98.8	22.0
4	**4d**	0	3–F	Me	91.9	213.0	98.9	4.8	99.3	2.7
5	**4e**	0	4–F	Me	87.6	426.0	98.4	10.0	99.1	4.5
6	**4f**	2	3–F	H	96.3	138.0	95.6	24.0	97.8	20.0
7	**4g**	2	4–F	H	96.5	90.0	97.7	22.0	97.9	22.0
8	**4h**	2	3–F	Me	94.3	170.0	98.7	16.0	98.6	14.0
9	**4i**	2	4–F	Me	96.9	173.0	97.6	22.0	95.0	12.0
10	**3**	1	3–F	Me	95.6	128.0	97.4	7.9	98.2	0.7

**Table 2:** Binding affinities of optically active pyrimidines **4d** and **4e** against 5-HT_2_ receptor subtypes

Entry	Comp.	*n*	F	R	5-HT_2A_		5-HT_2B_		5-HT_2C_	
					%Binding	*K*_i_ (nM)	%Binding	*K*_i_ (nM)	%Binding	*K*_i_ (nM)
1	(*R*,*R*)-**4d**	0	3–F	Me (*R*)	93.3	222.0	100.2	2.6	98.5	1.2
2	(*S*,*R*)-**4d**	0	3–F	Me (*S*)	82.4	475.0	95.2	67.0	97.7	14.0
3	(*R*,*R*)-**4e**	0	4–F	Me (*R*)	0.9	-	99.6	19.0	94.2	4.0
4	(*S*,*R*)-**4e**	0	4–F	Me (*S*)	64.8	1024.0	95.3	128.0	97.8	23.0

## Supplementary Materials

The supplementary materials are available online.
