# Supplementary material for: Identification of Optically Active Pyrimidine Derivatives as Selective 5-HT2C Modulators
Source: Molecules. 2017 Aug 26;22(9):1416. doi: 10.3390/molecules22091416 (PMC6151589; doi:10.3390/molecules22091416)
Supplement: Supplementary file 1 [file molecules-22-01416-s001.pdf]

Supplementary Information

Identification of optically active pyrimidine derivatives as selective 5-HT_{2C} modulators

Juhyeon Kim [1,2] , Hanbyeol Jo [3] , Hyunseung Lee [3] , Hyunah Choo [1,4] , Hak Joong Kim [2] , Ae Nim Pae [4,5] , Yong Seo Cho [1,4,*] and Sun-Joon Min [3,*]

[1] *Center for Neuro-Medicine, Korea Institute of Science and Technology (KIST), 5 Hwarangno 14-gil, Seongbuk-gu, Seoul, 02792, Republic of Korea*

[2] *Department of Chemistry, Korea University, Seoul, 02841, Republic of Korea*

[3] *Department of Chemical & Molecular Engineering/Applied Chemistry, Hanyang University, Ansan, Gyeonggi-do, 15588, Republic of Korea*

[4] *Department of Biological Chemistry, Korea University of Science and Technology (UST), 217 Gajungro, Yuseong-gu, Daejeon, 34113, Republic of Korea*

[5] *Convergence Research Center for Diagnosis, Treatment and Care System of Dementia, KIST, Seoul 02792, Republic of Korea*

Email: sjmin@hanyang.ac.kr;ys4049@kist.re.kr

Table of Contents

1. Determination of optical purities of compounds (*R*)/(*S*)-**5d** and **5e** S2

2. Radioligands and reference compounds for binding assay S4

3. Binding affinity of (*R*,*R*)/(*S*,*R*)-**4d** and **4e** against 5-HT receptor subtypes S5

4. NMR spectral data of pyrimidine derivatives **4a**-**4i** S6

1. Determination of optical purities of compounds (*R*)/(*S*)-**5d** and **5e**

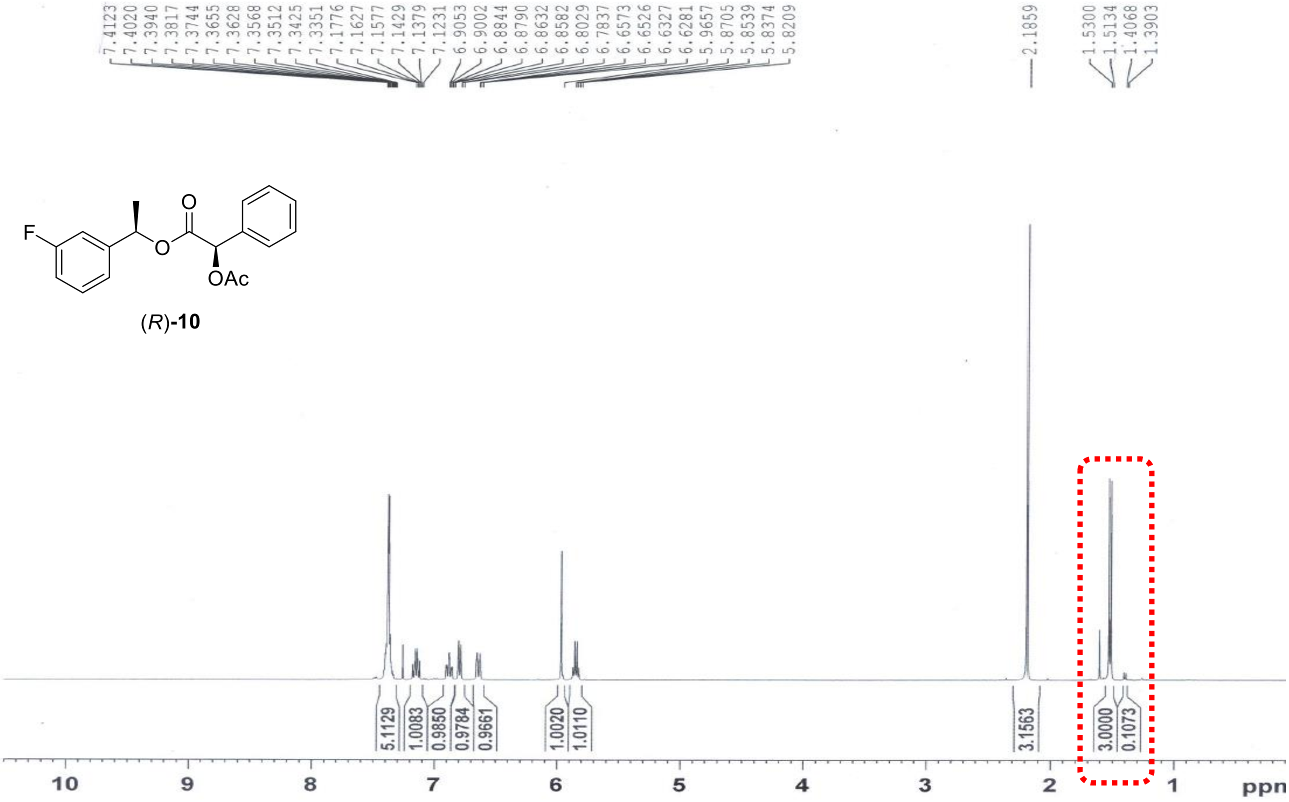

^{1}H NMR Spectrum of Compound (*R*)-**10** (from (*R*)-**5d**) (400 MHz)

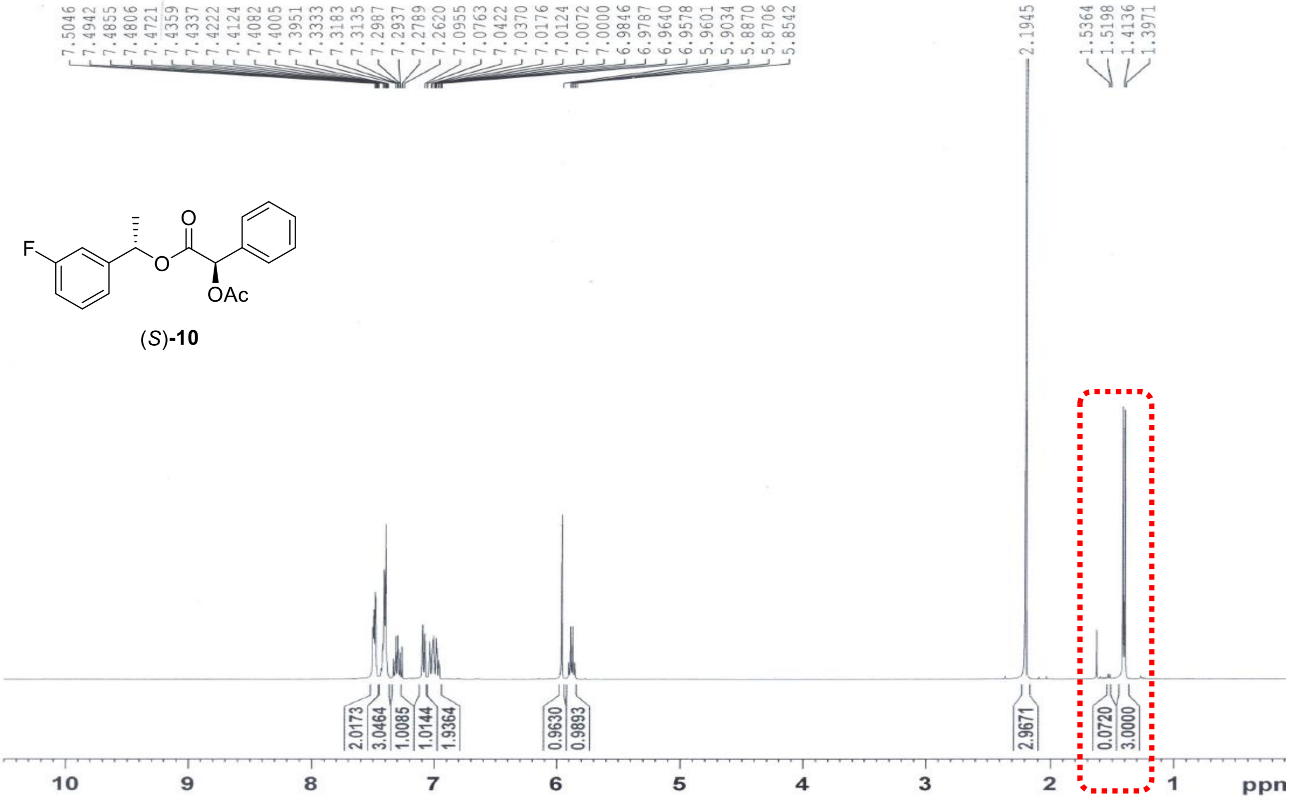

^{1}H NMR Spectrum of Compound (*S*)-**10** (from (*S*)-**5d**) (400 MHz)

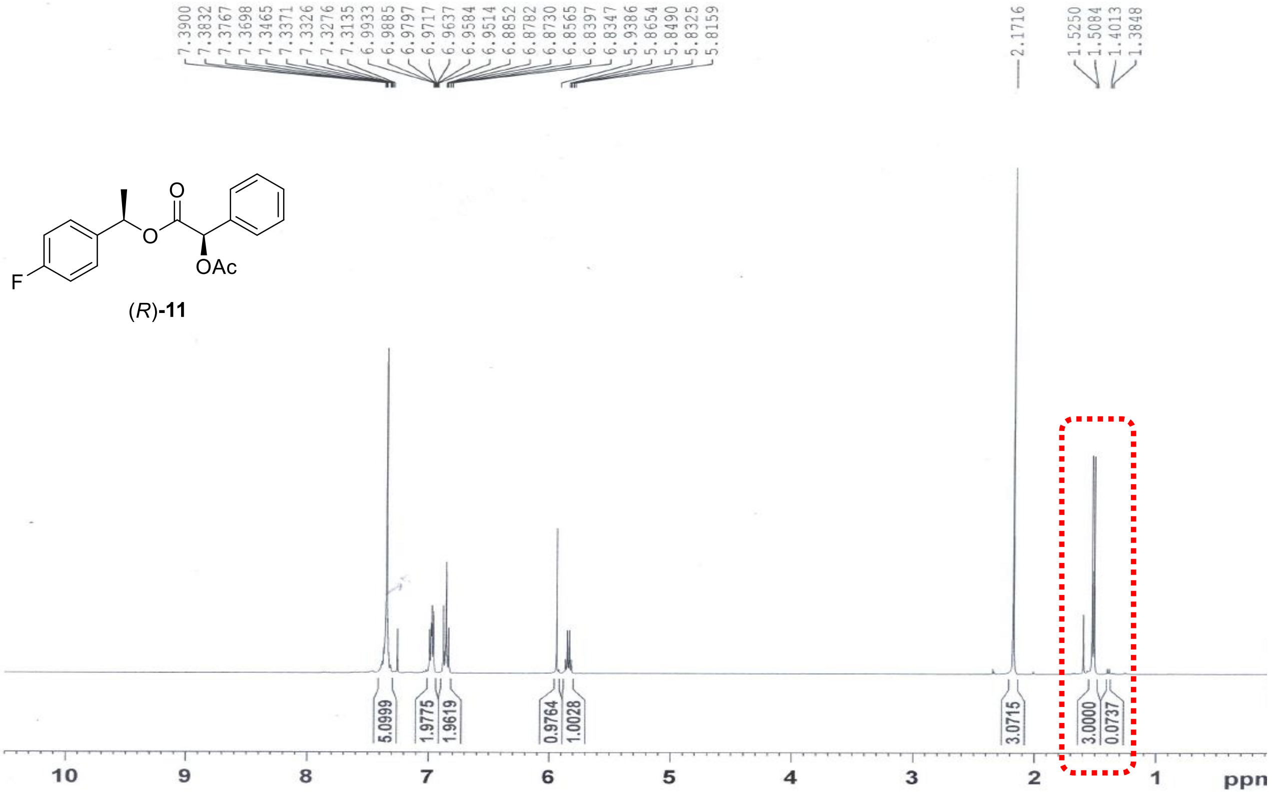

^{1}H NMR Spectrum of Compound (*R*)-**11** (from (*R*)-**5e**) (400 MHz)

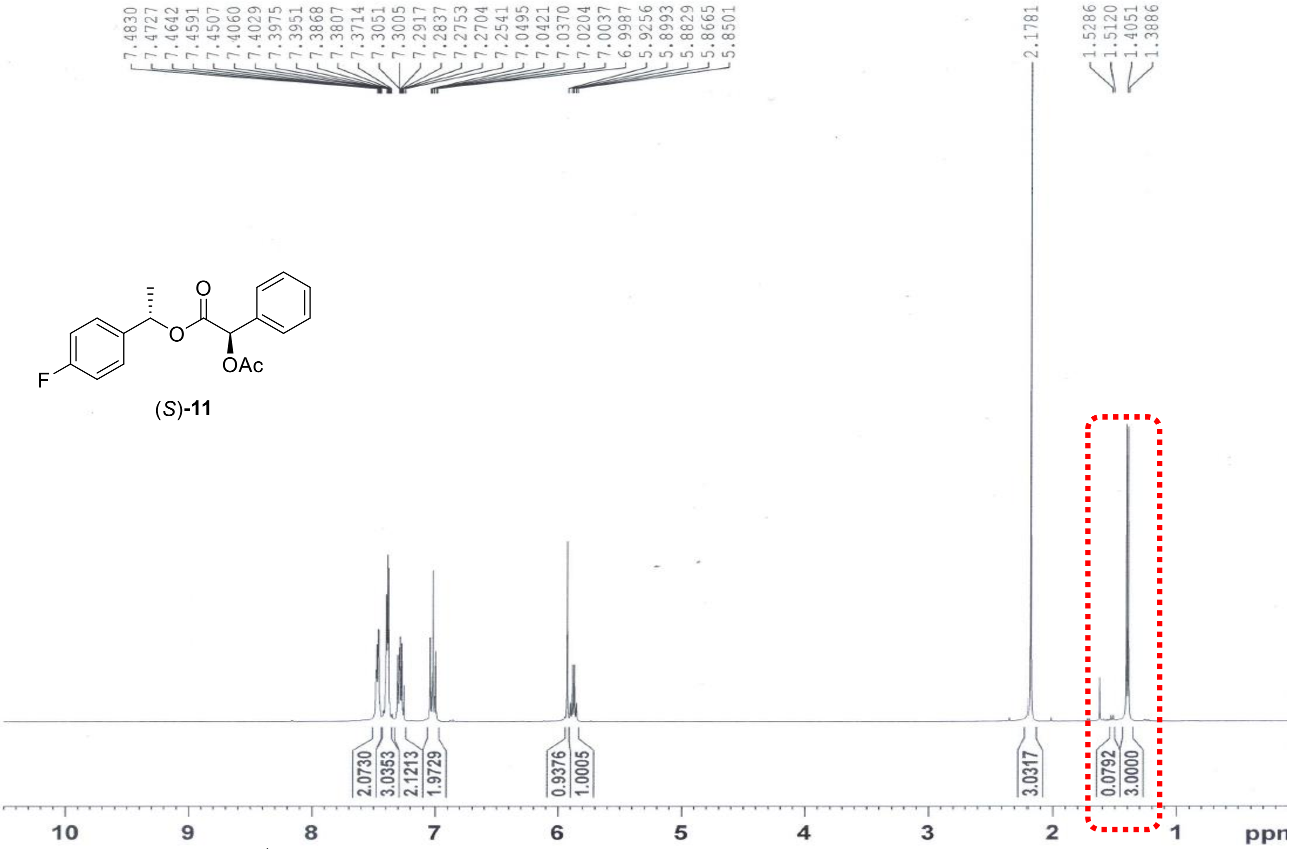

^{1}H NMR Spectrum of Compound (*S*)-**11** (from (*S*)-**5e**) (400 MHz)

2. Radioligands and reference compounds for binding assay

Table S1. A list of 5-HT receptor radioligands and reference compounds for binding assay.

**Receptor subtype**	**Radioligand**	**Reference compound**
**1A**	[^{3}H]8-OH-DPAT	Methysergide
**1B**	[^{3}H]GR125743	Ergotamine
**1D**	[^{3}H]GR125743	Ergotamine
**1E**	[^{3}H]5-HT	5-HT
**2A**	[^{3}H]Ketanserin	Chlorpromazine
**2B**	[^{3}H]LSD	5-HT
**2C**	[^{3}H]Mesulergine	Chlorpromazine
**3**	[^{3}H]LY278584	LY278584
**5A**	[^{3}H]LSD	Ergotamine
**6**	[^{3}H]LSD	Chlorpromazine
**7**	[^{3}H]LSD	Chlorpromazine

3. Binding affinity of (*R*,*R*)/(*S*,*R*)-**4d** and **4e** against 5-HT receptor subtypes

Table S2. Binding affinity evaluation of compounds (*R*,*R*)/(*S*,*R*)-**4d** and **4e** against 5-HT receptor subtypes[a]

compd.	5-HT subtypes	**1A**	**1B**	**1D**	**1E**	**2A**	**2B**	**2C**	**3**	**5A**	**6**	**7**
(*R*,*R*)-**4d**	% binding at 10 µM	78.0	27.7	-6.8	67.5	93.3	100.2	**98.5**	89.4	28.1	95.7	84.4
	*K*i (nM)	98.0	-[b]	-[b]	1161.0	222.0	2.6	**1.2**	242.0	-[b]	57.0	444.0
(*S*,*R*)-**4d**	% binding at 10 µM	67.7	3.0	10.3	29.5	82.4	95.2	**97.7**	78.6	22.8	91.6	56.9
	*K*i (nM)	806.0	-[b]	-[b]	-[b]	475.0	67.0	**14.0**	501.0	-[b]	70.0	766.0
(*R*,*R*)-**4e**	% binding at 10 µM	10.0	6.4	-7.7	42.7	0.9	99.6	**94.2**	87.8	26.7	97.4	87.6
	*K*i (nM)	-[b]	-[b]	-[b]	-[b]	-[b]	19.0	**4.0**	242.0	-[b]	17.0	236.0
(*S*,*R*)-**4e**	% binding at 10 µM	67.3	17.9	40.4	-[c]	64.8	95.3	**97.8**	70.1	5.2	86.1	64.8
	*K*i (nM)	1117.0	-[b]	-[b]	-[b]	1024.0	128.0	**23.0**	1000.0	-[b]	116.0	946.0
**3** (ref)	% binding at 10 µM	89.0	55.1	78.3	65.6	95.6	97.4	**98.2**	75.0	37.4	93.8	94.7
	*K*i (nM)	353.0	1780.0	542.0	1160.0	128.0	7.9	**0.7**	241.0	-[b]	43.0	84.0

[a] 5-HT receptor binding was determined by competitive binding assay using radioligands and reference compounds in Table S1. [b] Not determined due to low %binding. [c] Not determined

4. NMR spectral data of pyrimidine derivatives **4a**-**4i** and (*R*,*R*)/(*S*,*R*)-**4d** and **4e**

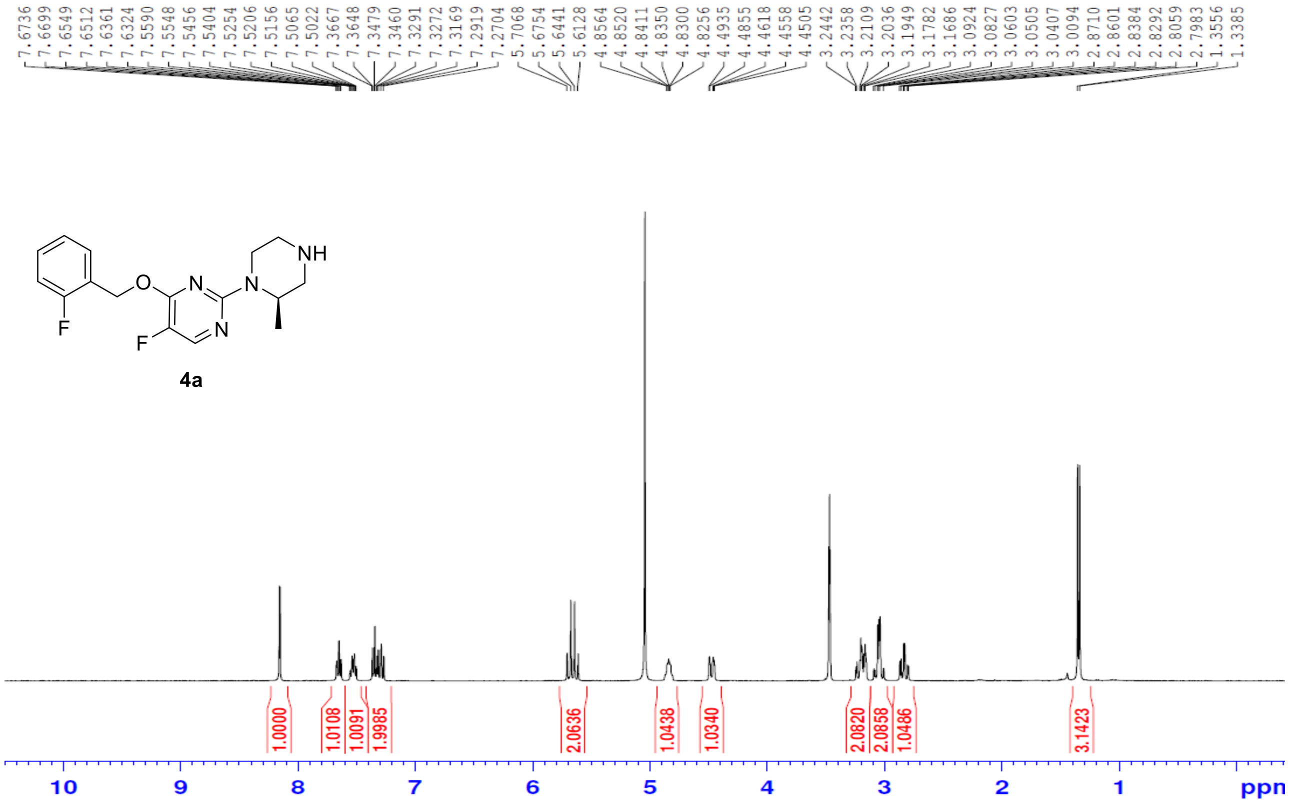

^{1}H NMR Spectrum of Compound **4a** (400 MHz)

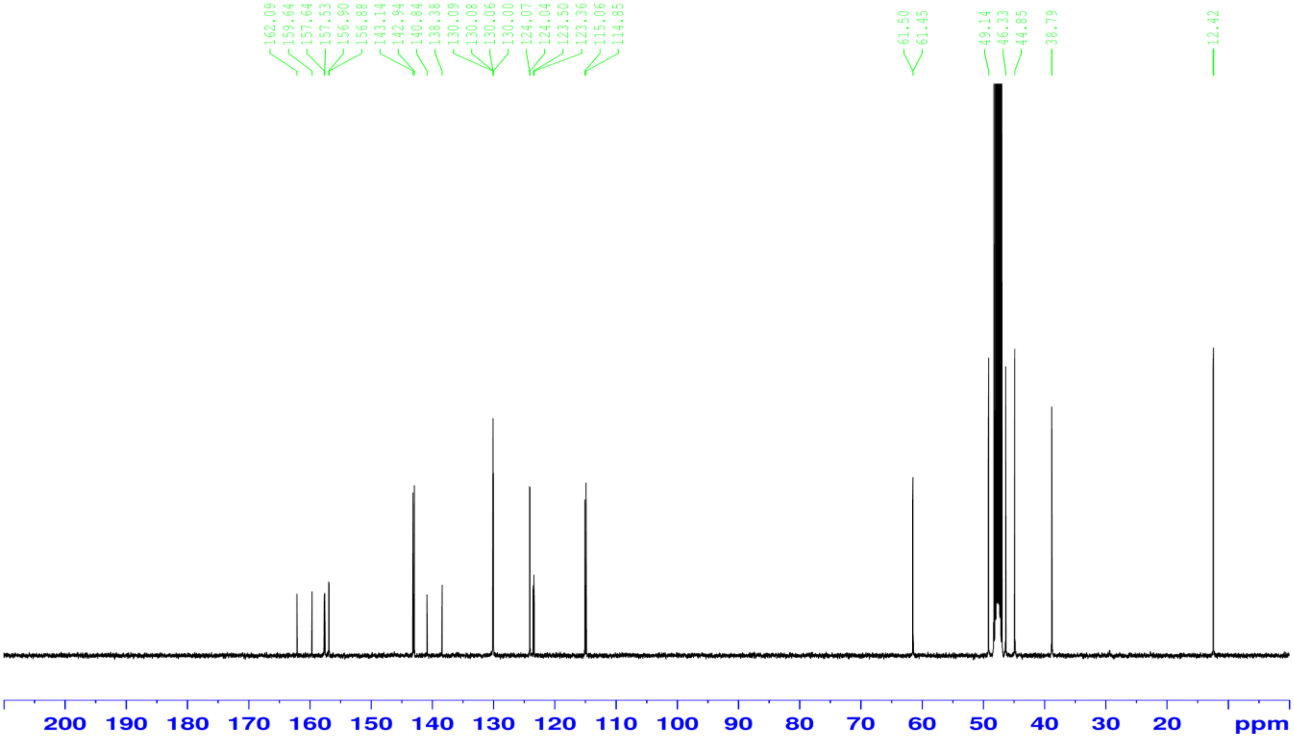

^{13}C NMR Spectrum of Compound **4a** (100 MHz)

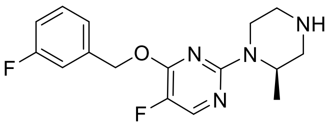

4b

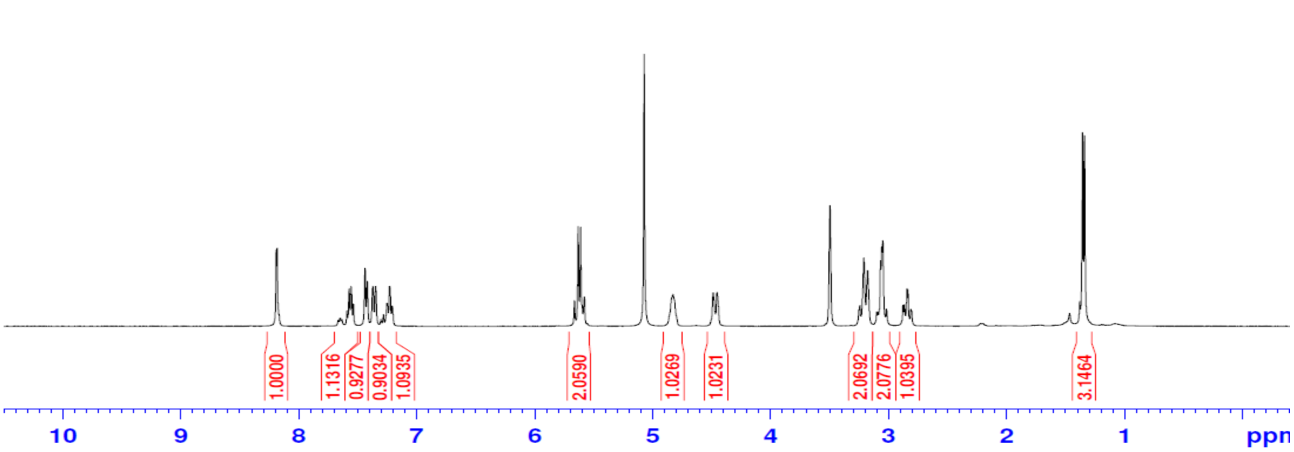

^{1}H NMR Spectrum of Compound **4b** (400 MHz)

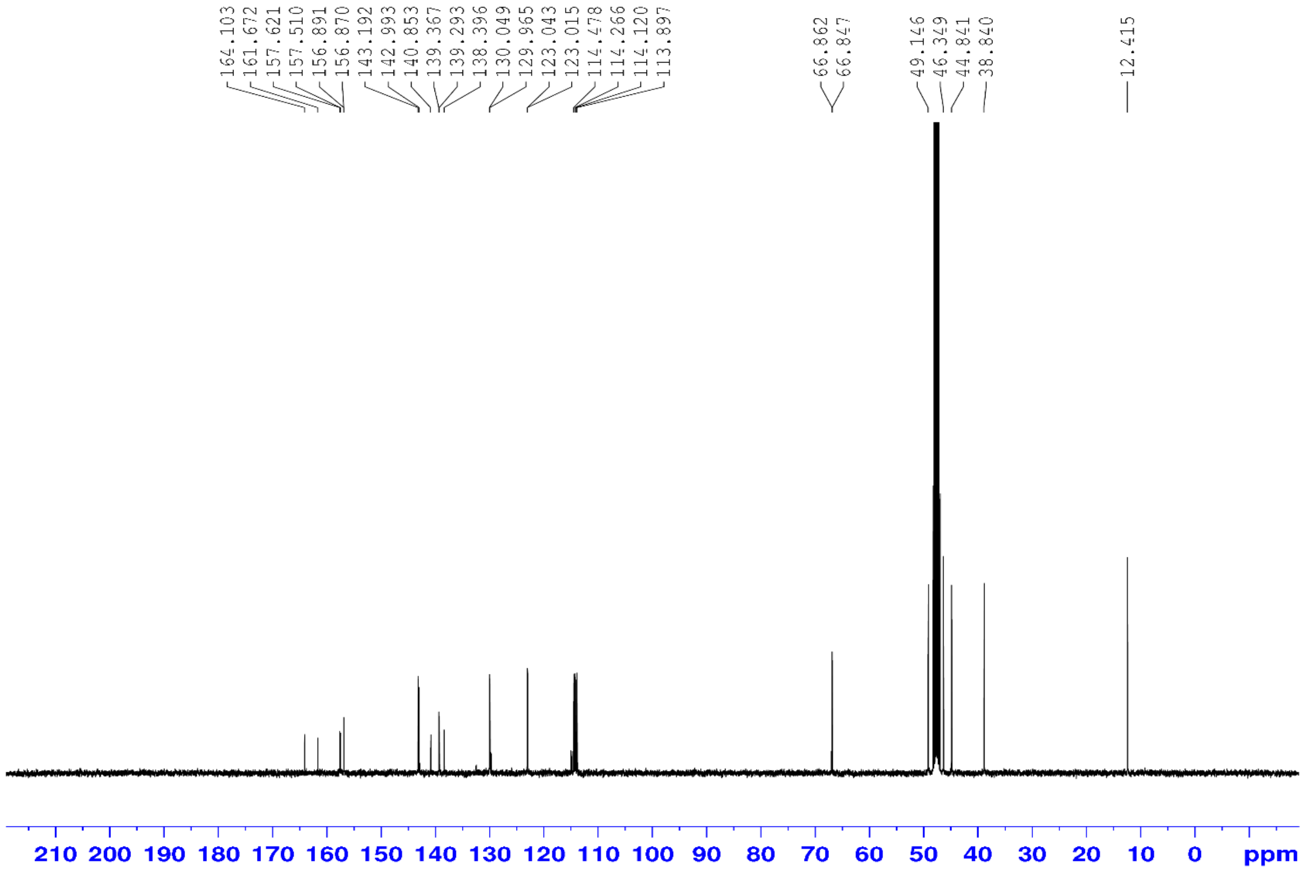

^{13}C NMR Spectrum of Compound **4b** (100 MHz)

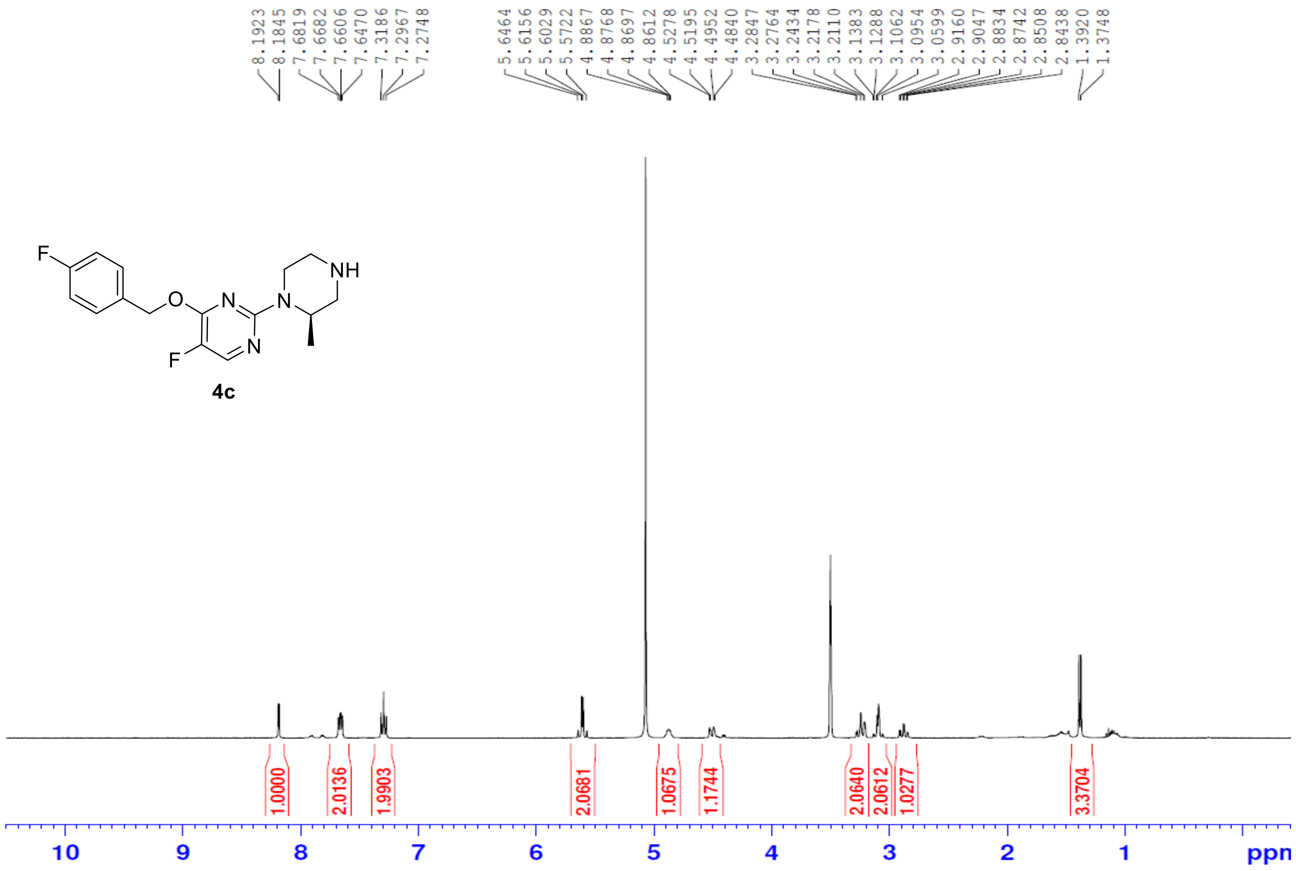

^{1}H NMR Spectrum of Compound **4c** (400 MHz)

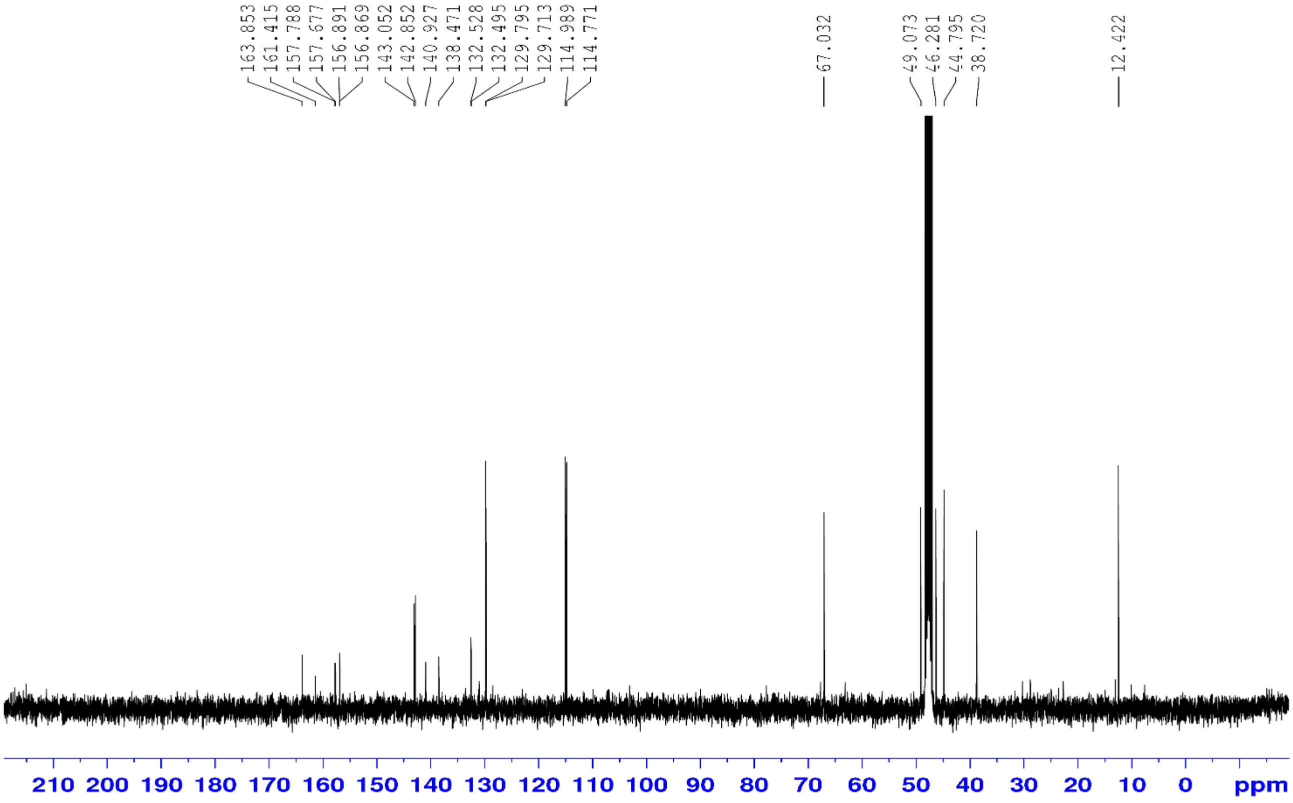

^{13}C NMR Spectrum of Compound **4c** (100 MHz)

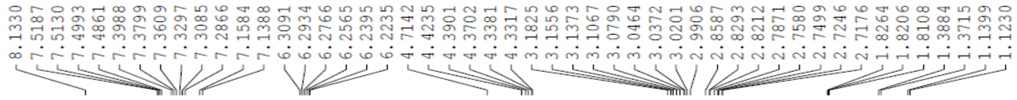

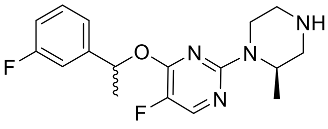

4d

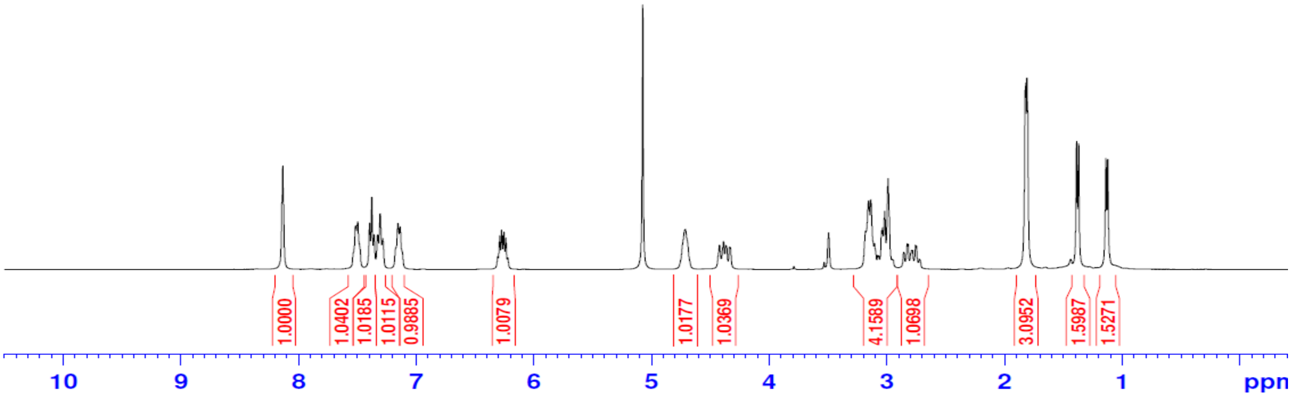

^{1}H NMR Spectrum of Compound **4d** (400 MHz)

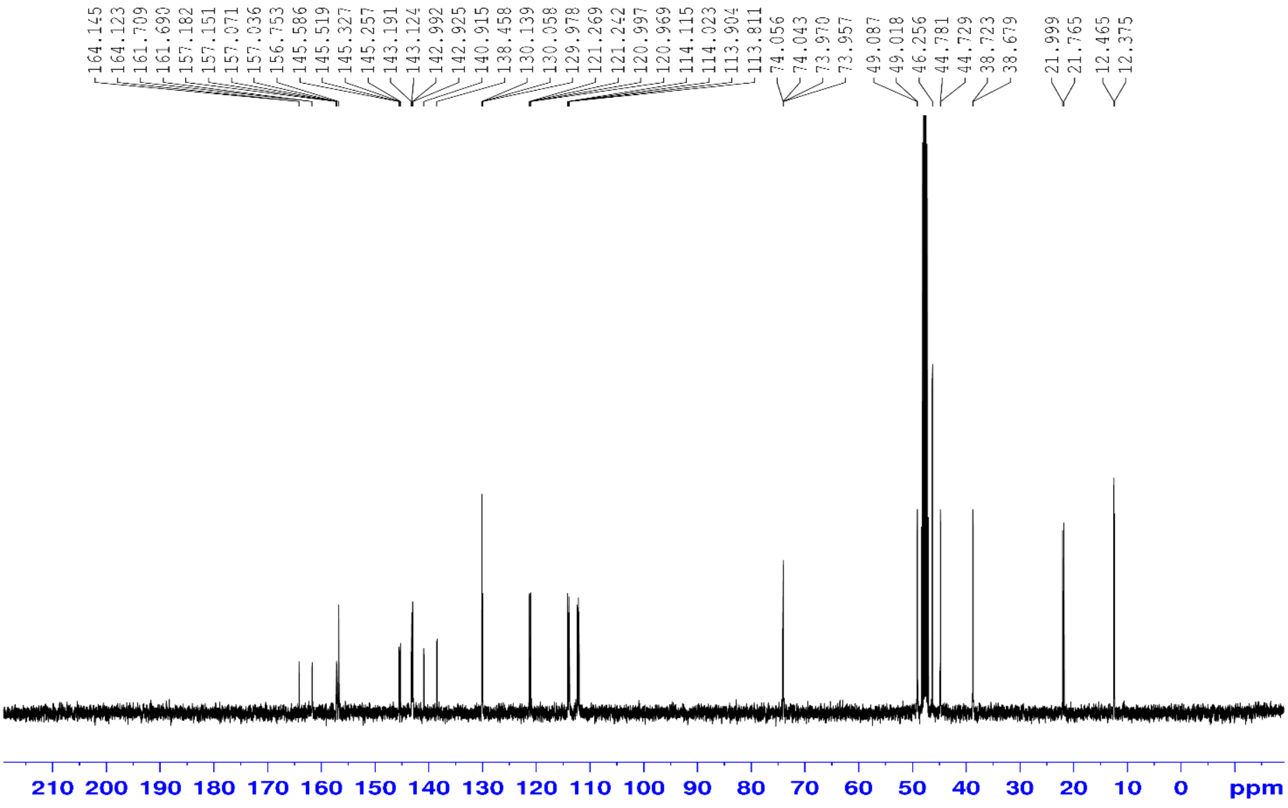

^{13}C NMR Spectrum of Compound **4d** (100 MHz)

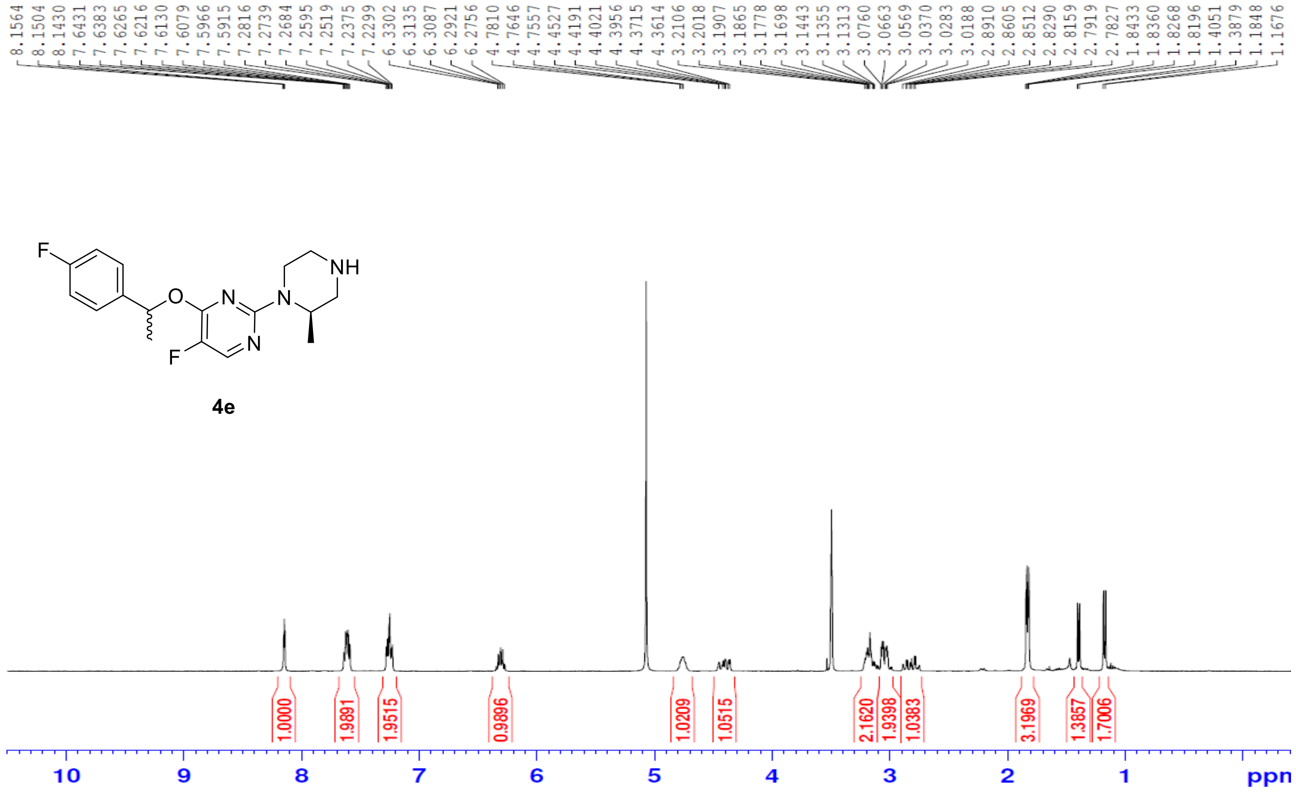

^{1}H NMR Spectrum of Compound **4e** (400 MHz)

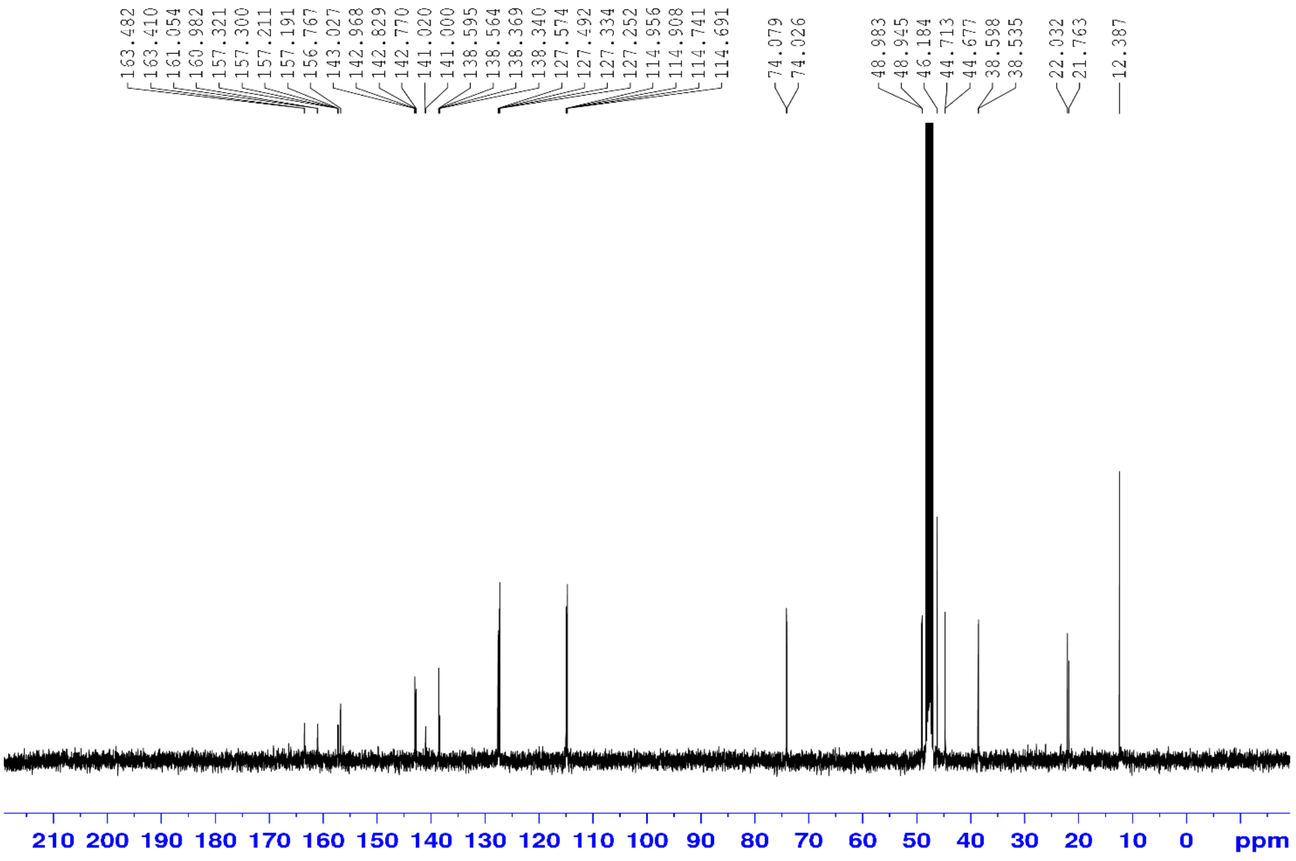

^{13}C NMR Spectrum of Compound **4e** (100 MHz)

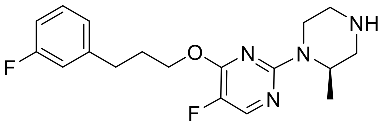

4f

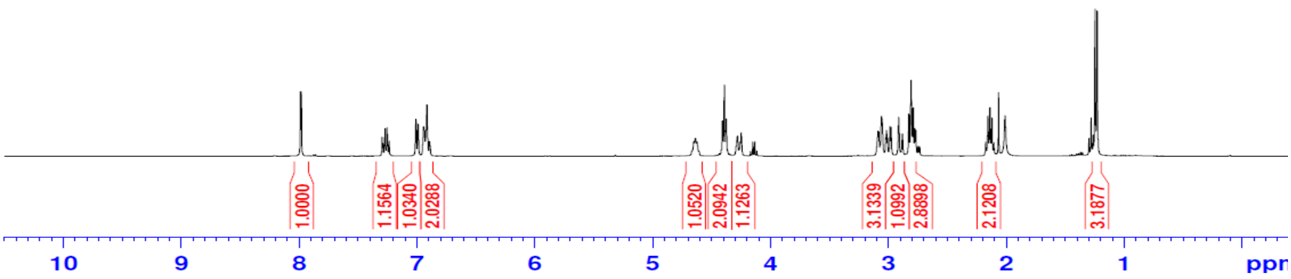

^{1}H NMR Spectrum of Compound **4f** (400 MHz)

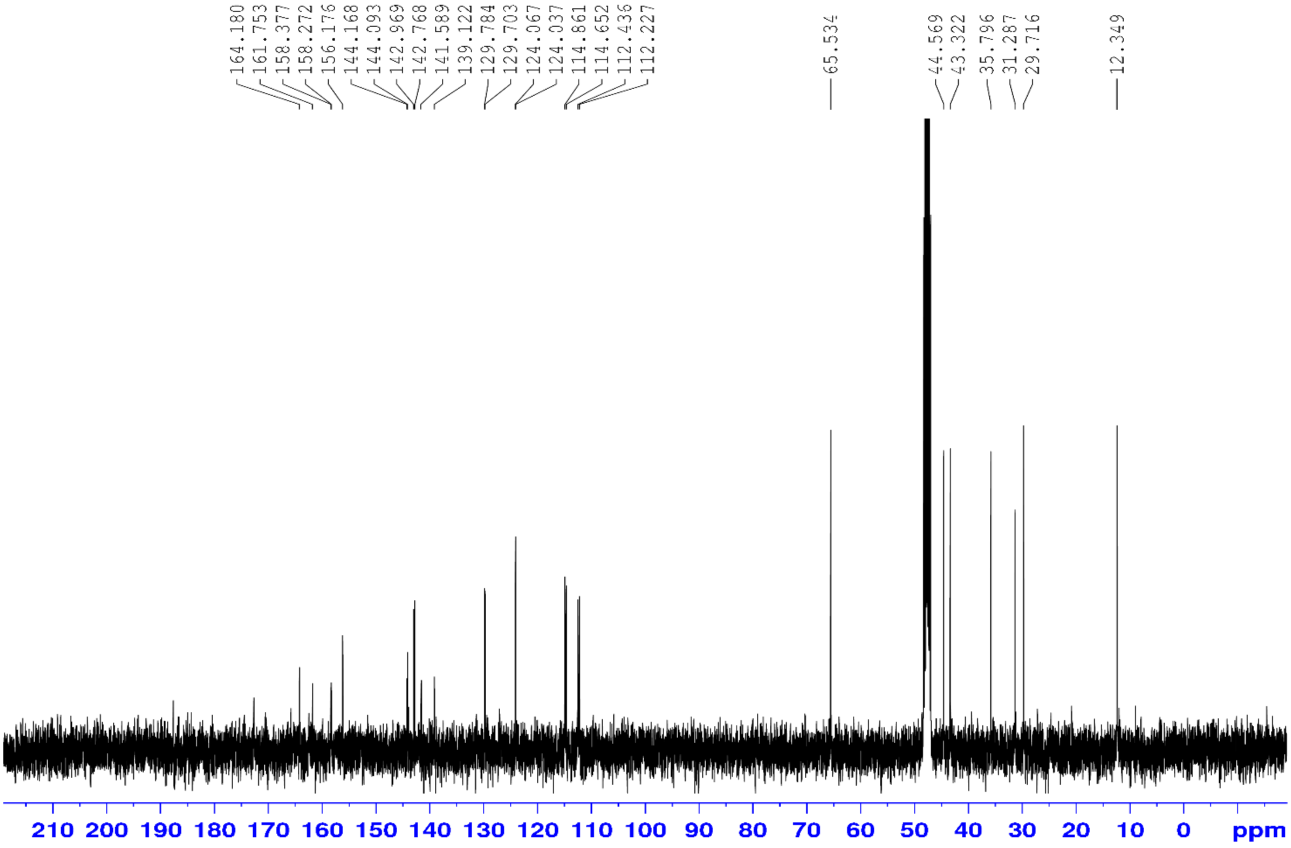

^{13}C NMR Spectrum of Compound **4f** (100 MHz)

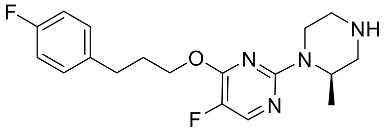

4g

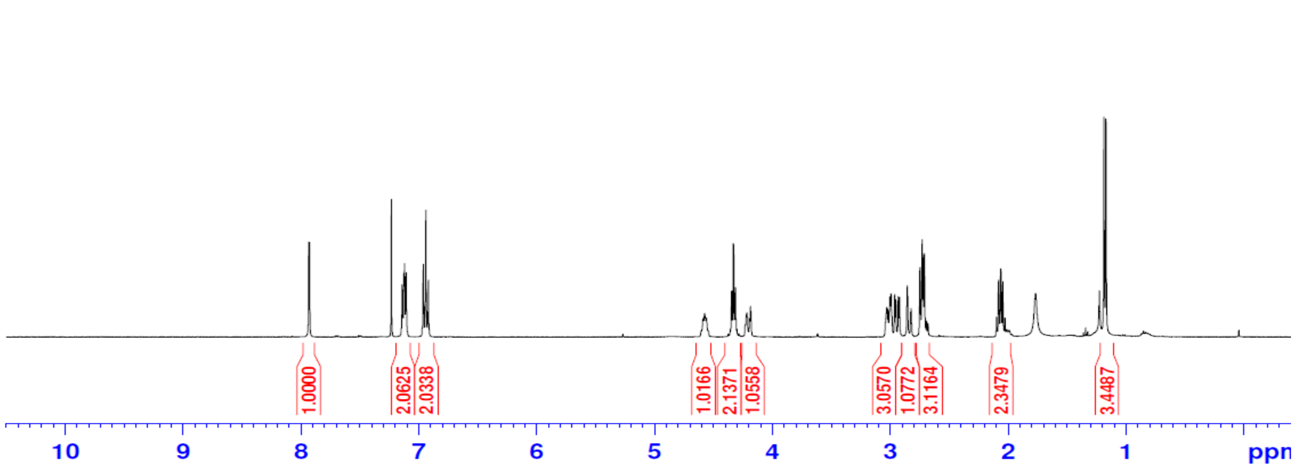

^{1}H NMR Spectrum of Compound **4g** (400 MHz)

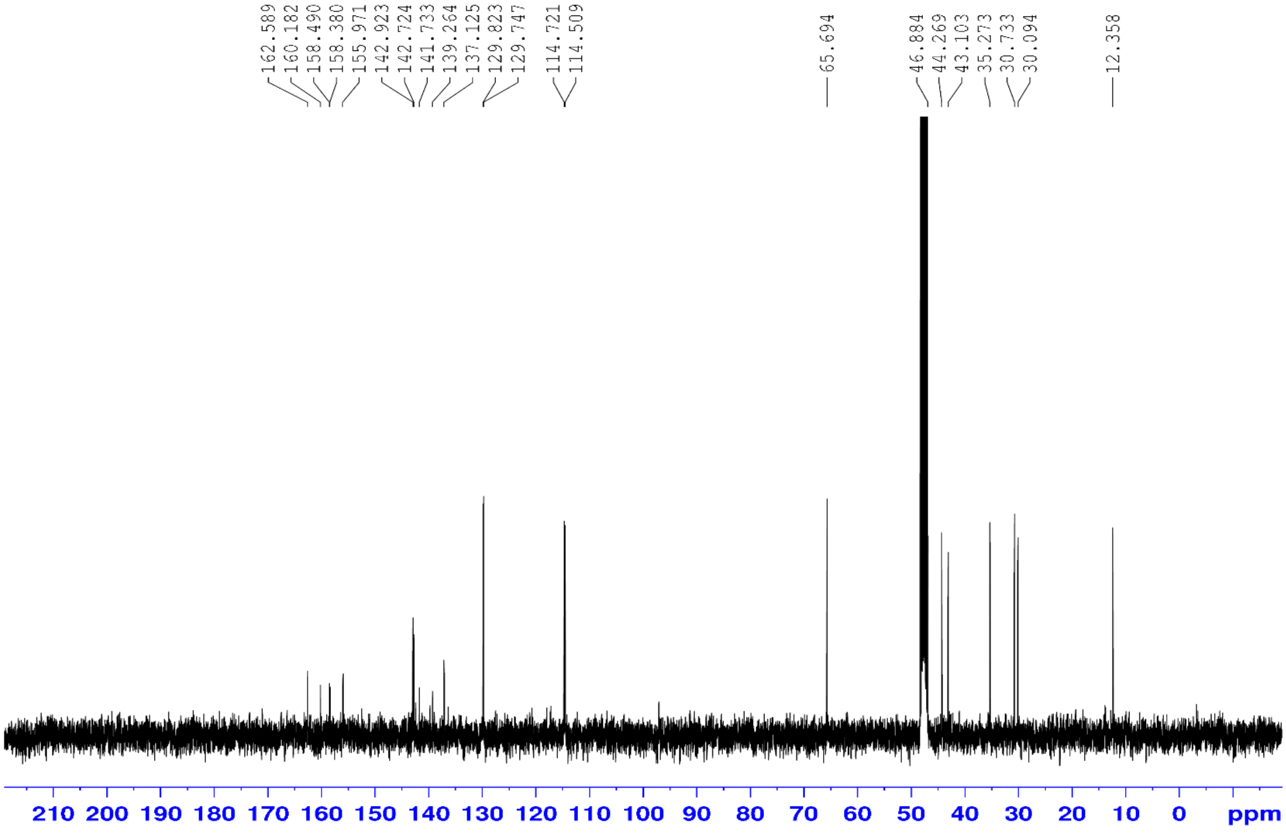

^{13}C NMR Spectrum of Compound **4g** (100 MHz)

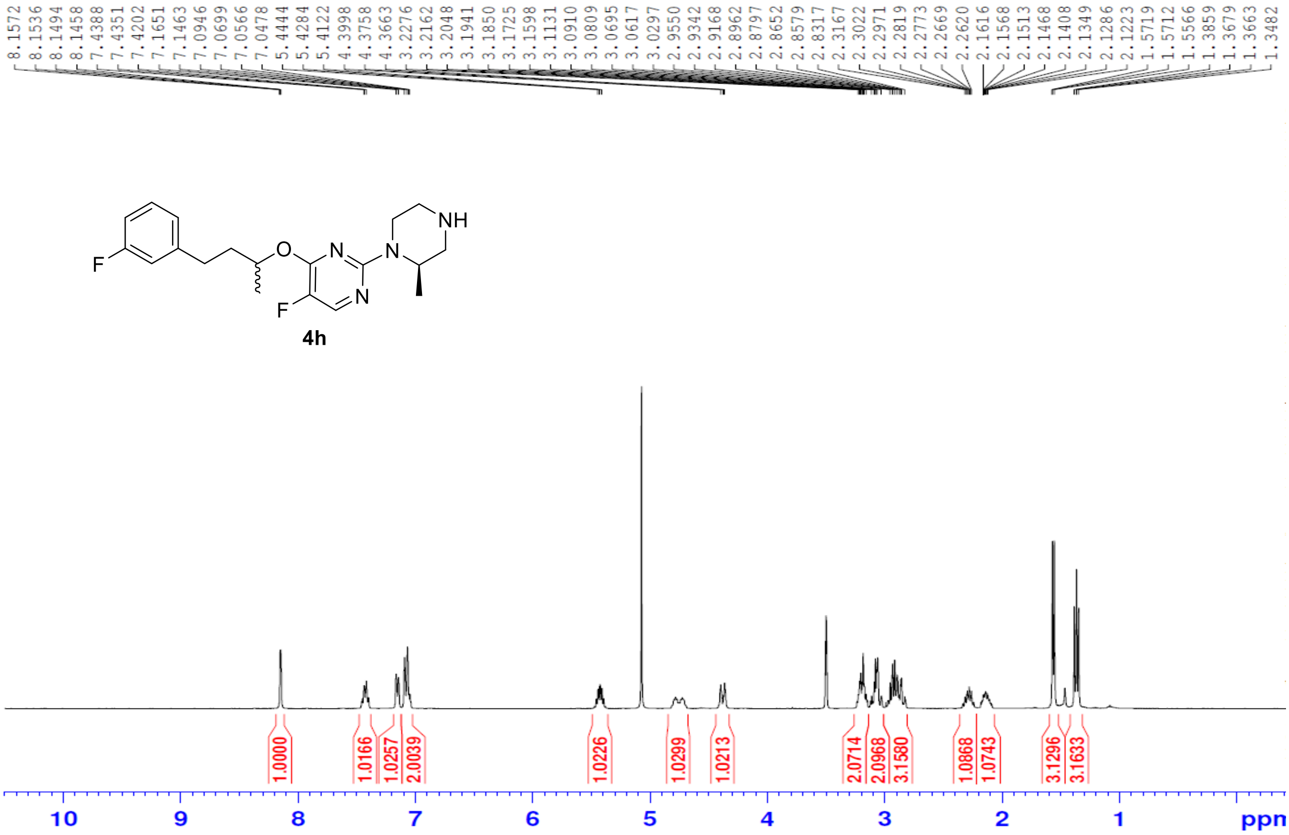

^{1}H NMR Spectrum of Compound **4h** (400 MHz)

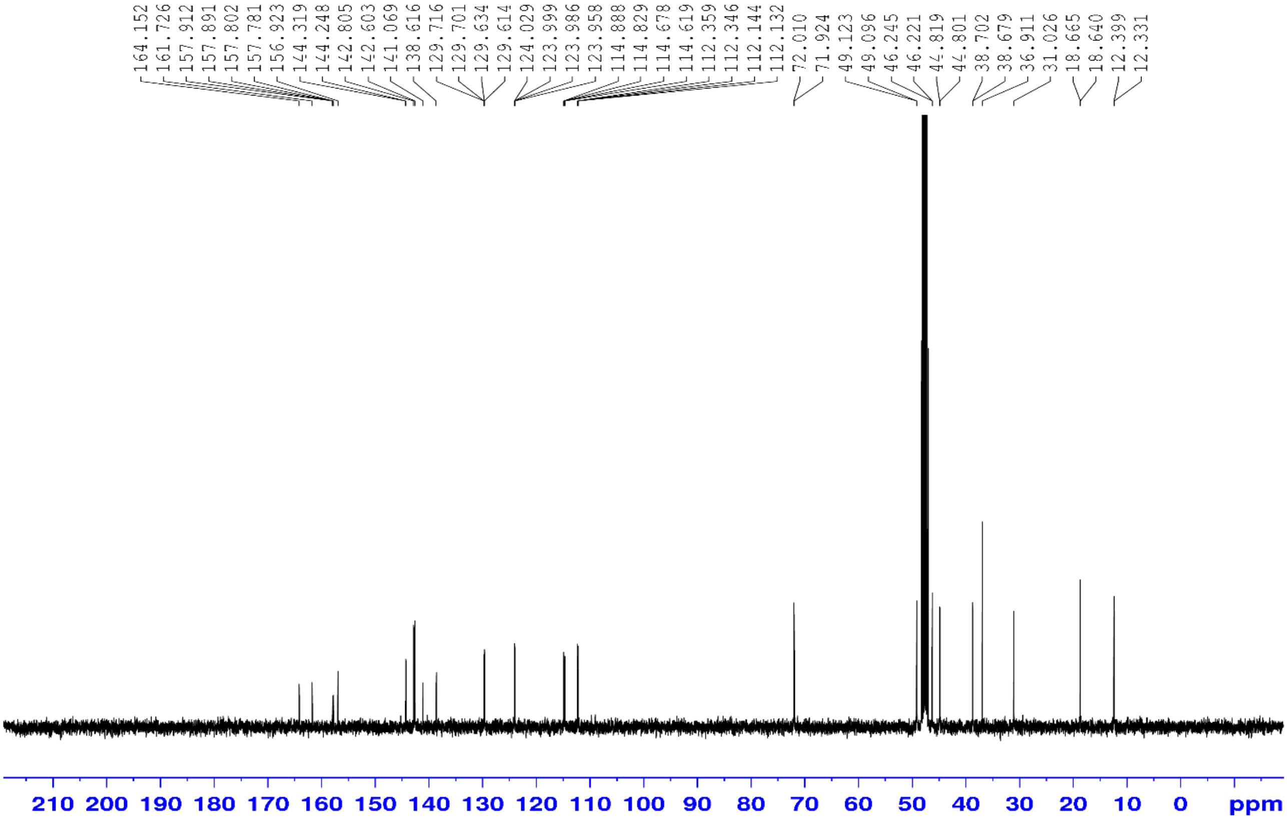

^{13}C NMR Spectrum of Compound **4h** (100 MHz)

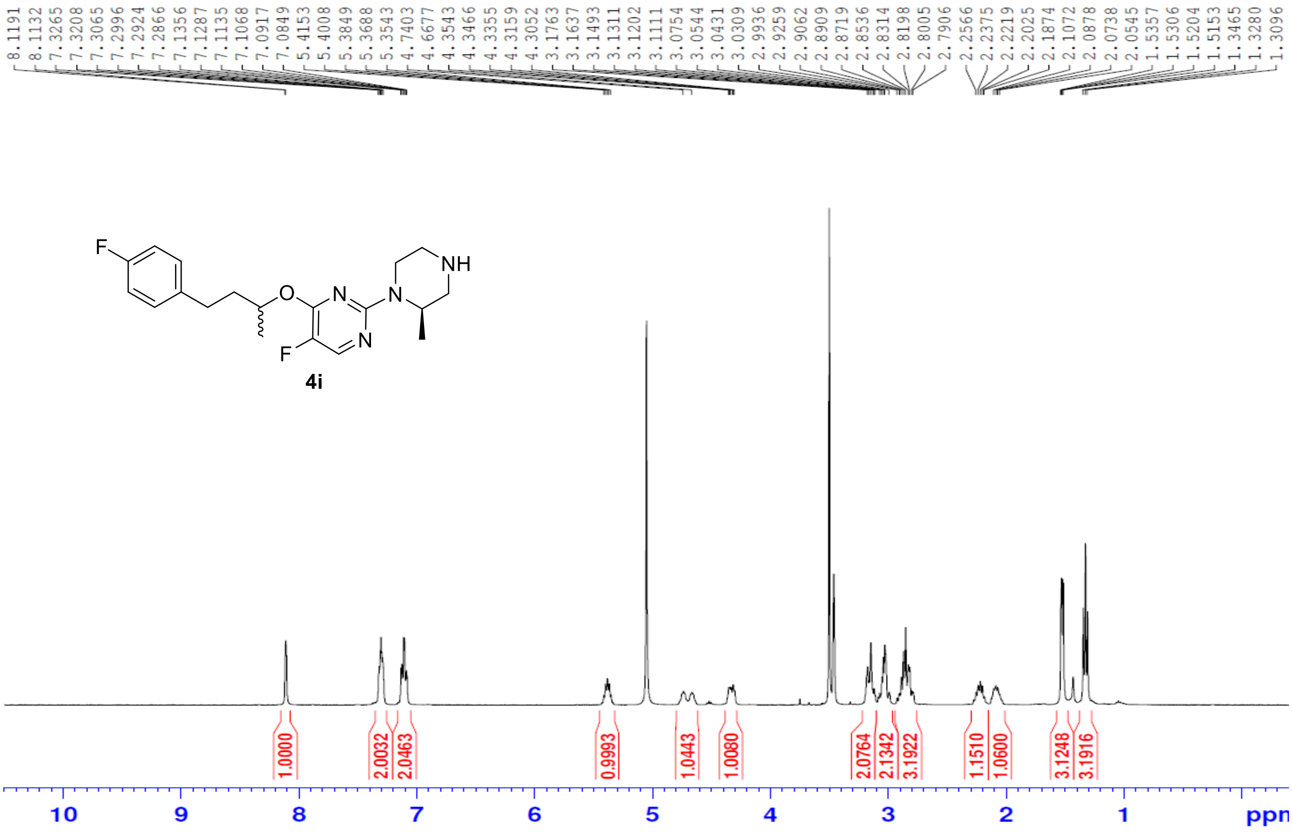

^{1}H NMR Spectrum of Compound **4i** (400 MHz)

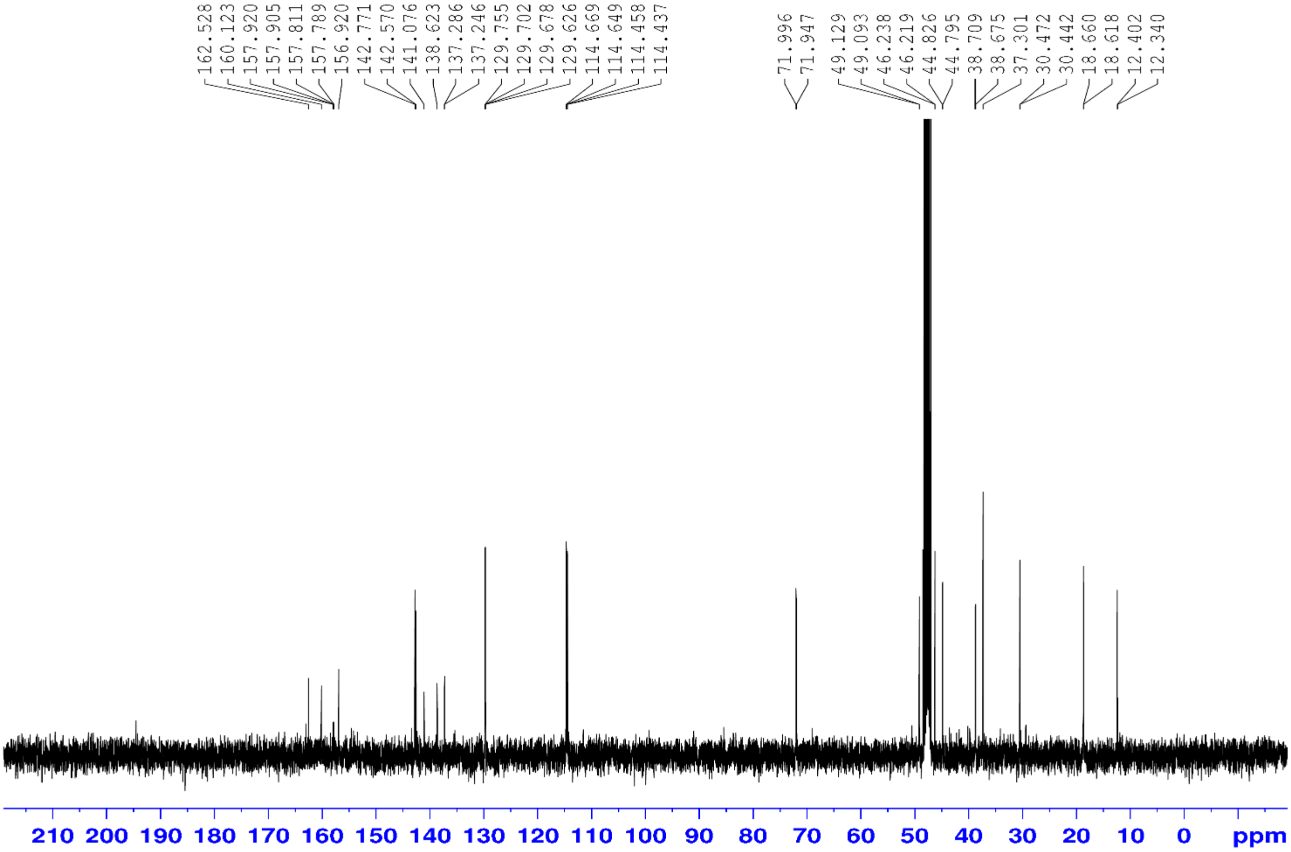

^{13}C NMR Spectrum of Compound **4i** (100 MHz)

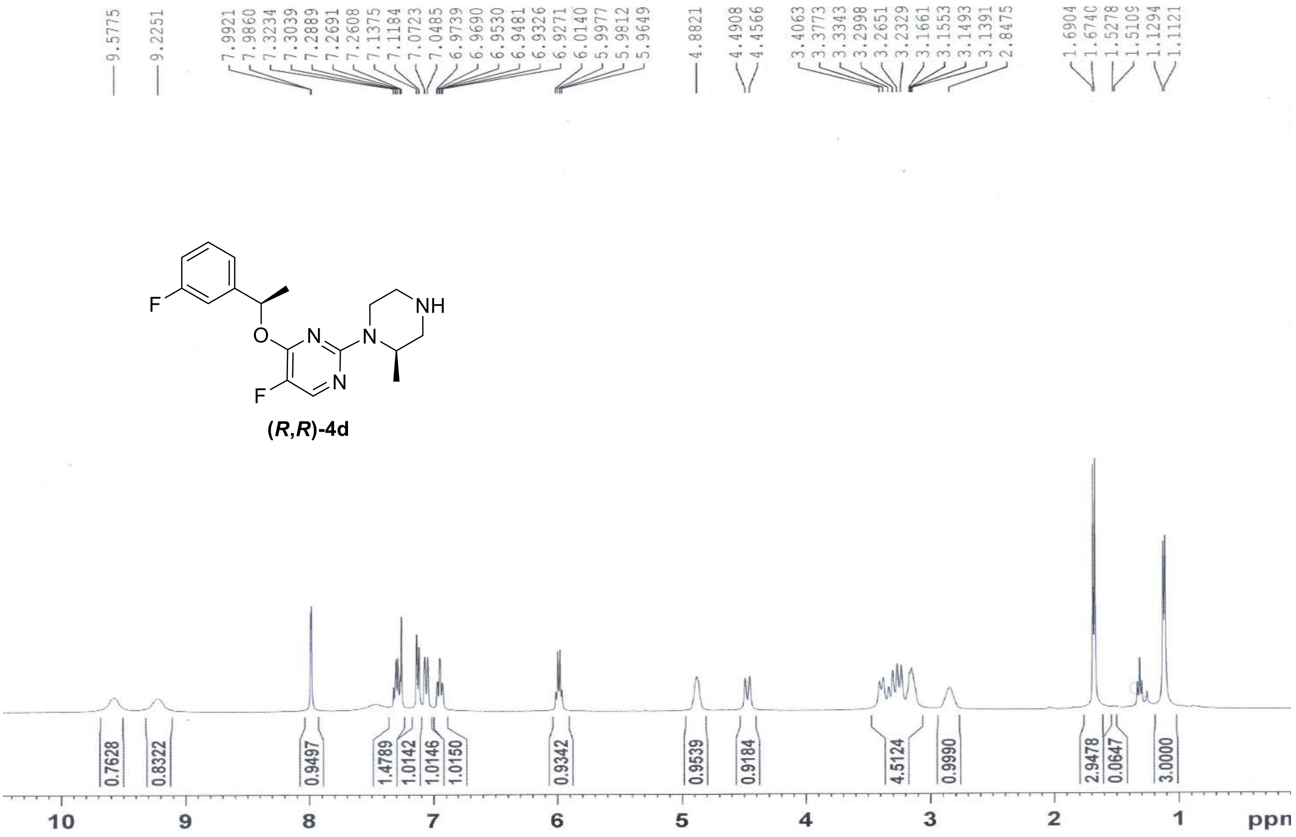

^{1}H NMR Spectrum of Compound (*R*,*R*)-**4d** (400 MHz)

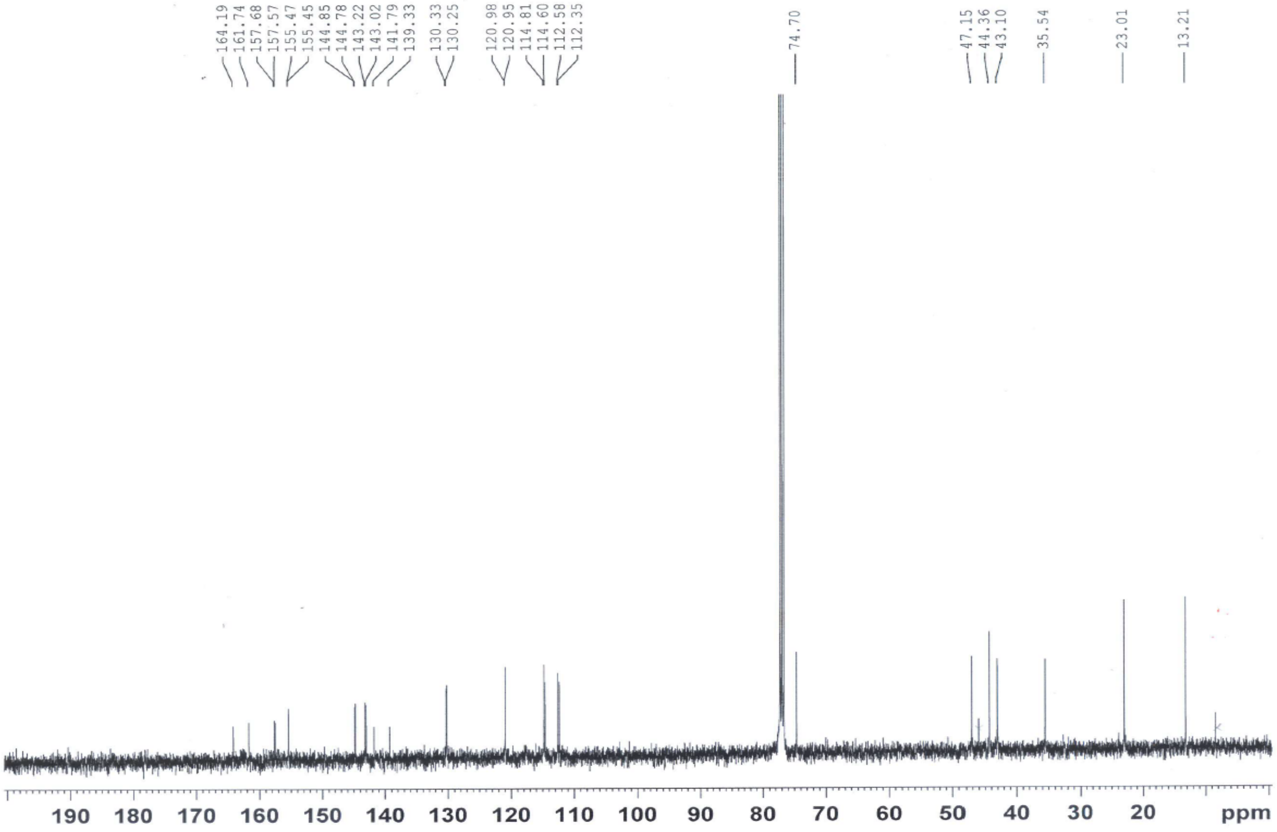

^{13}C NMR Spectrum of Compound (*R*,*R*)-**4d** (100 MHz)

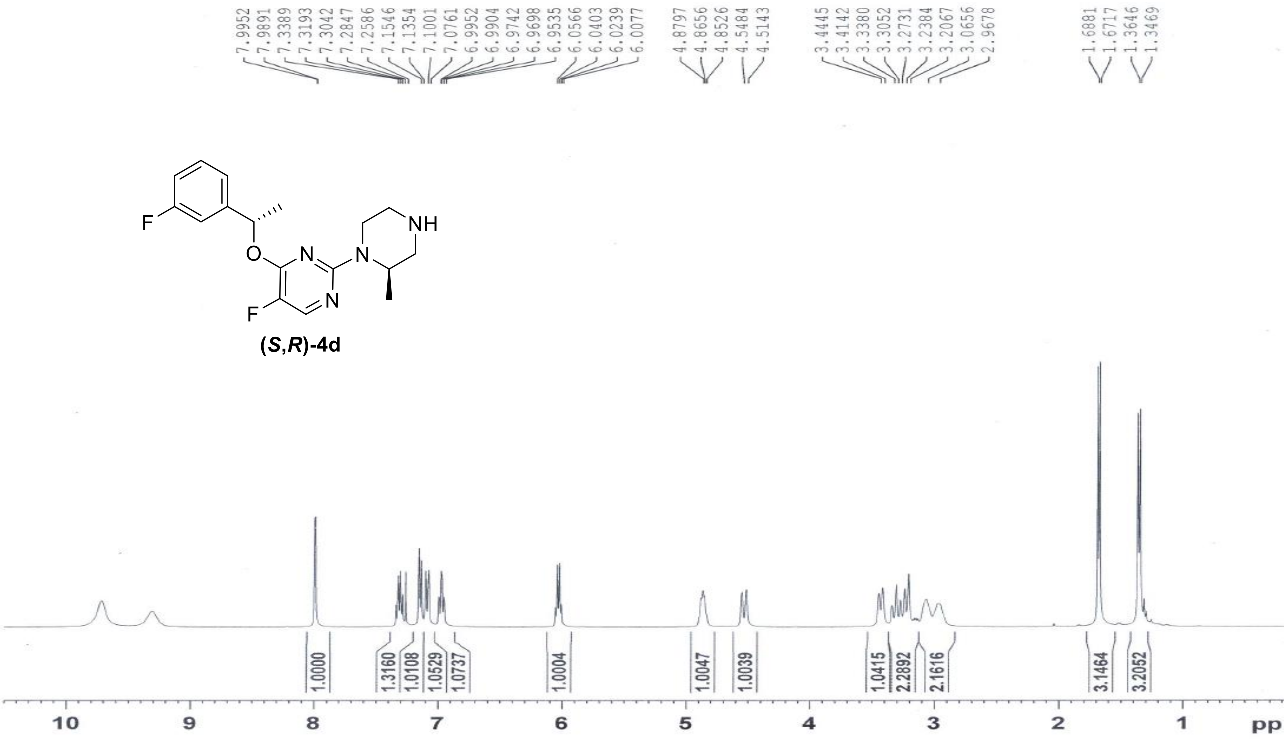

^{1}H NMR Spectrum of Compound (*S*,*R*)-**4d** (400 MHz)

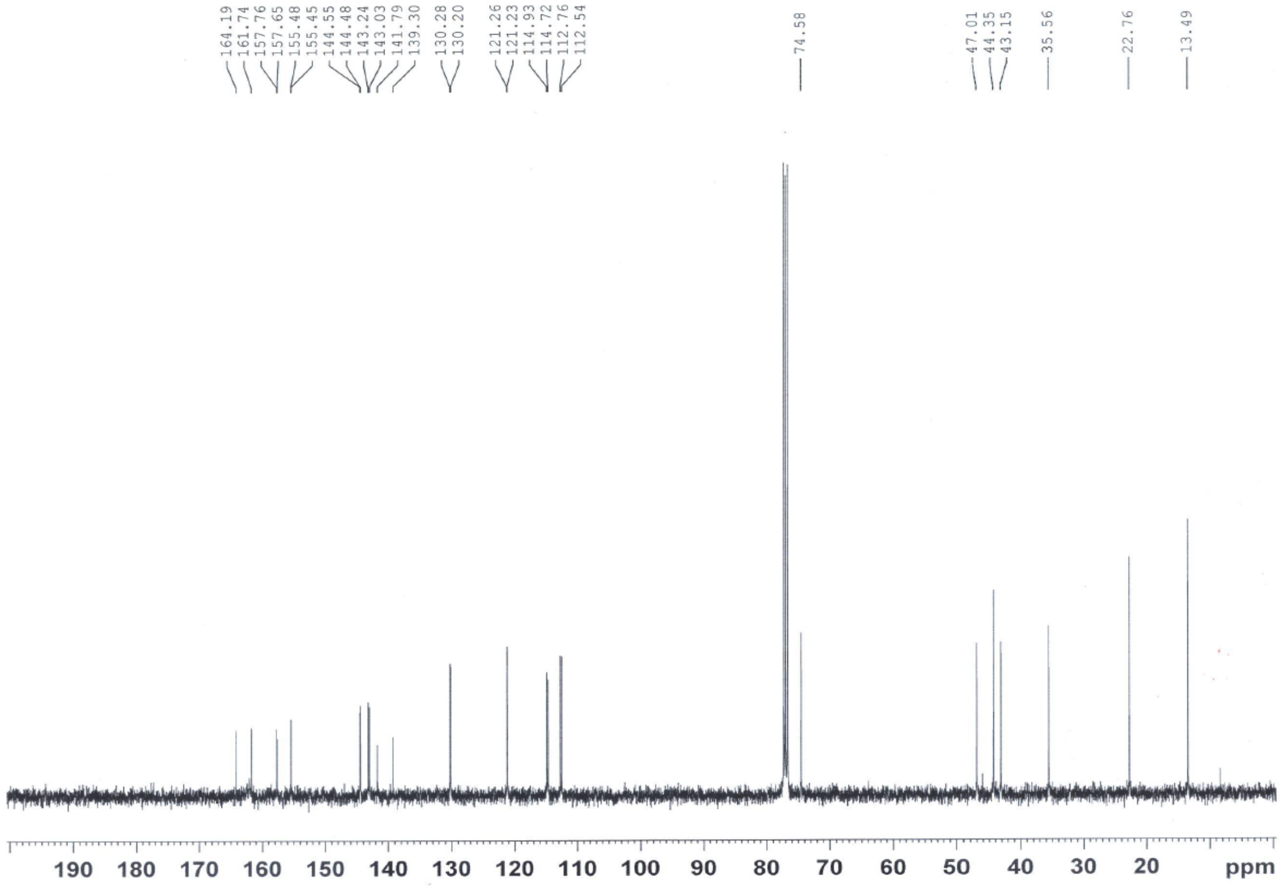

^{13}C NMR Spectrum of Compound (*S*,*R*)-**4d** (100 MHz)

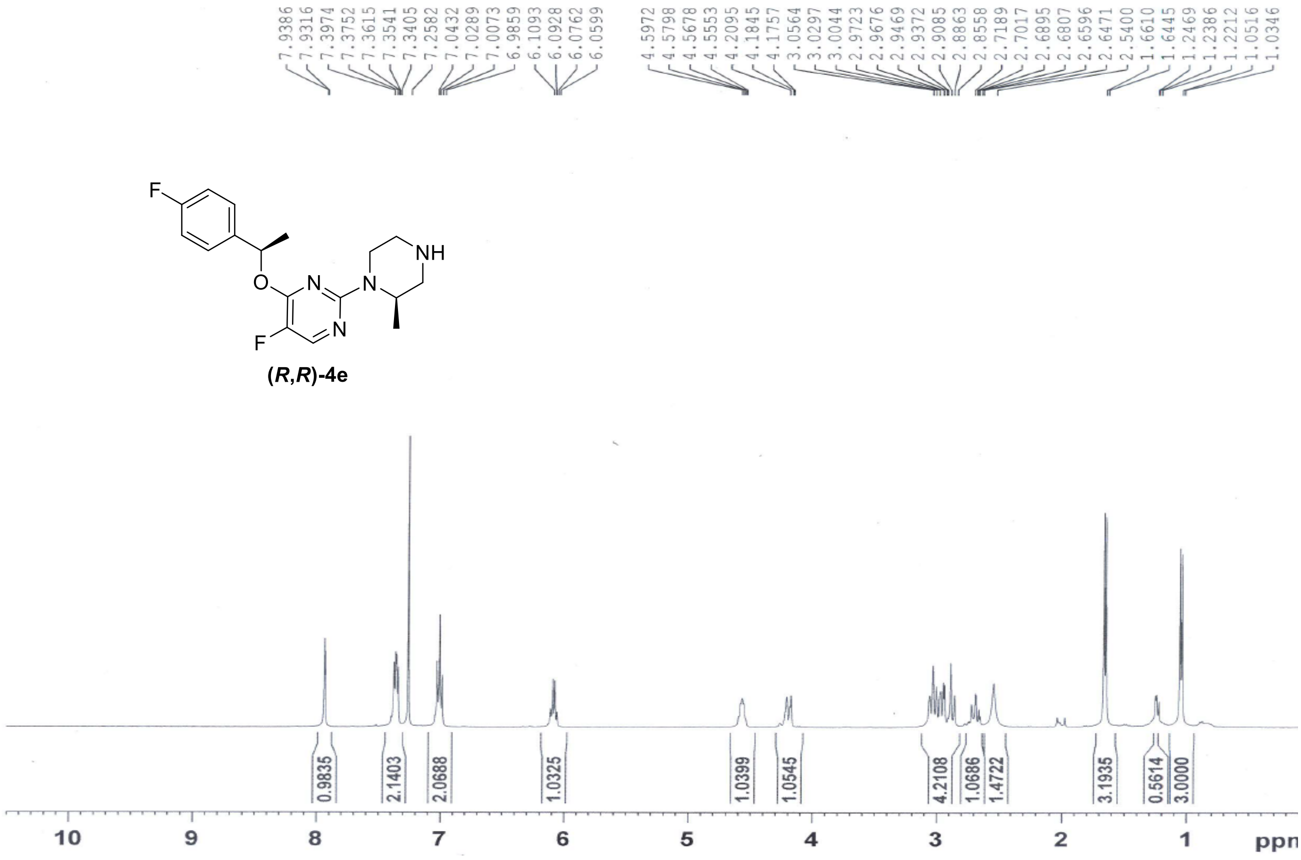

^{1}H NMR Spectrum of Compound (*R*,*R*)-**4e** (400 MHz)

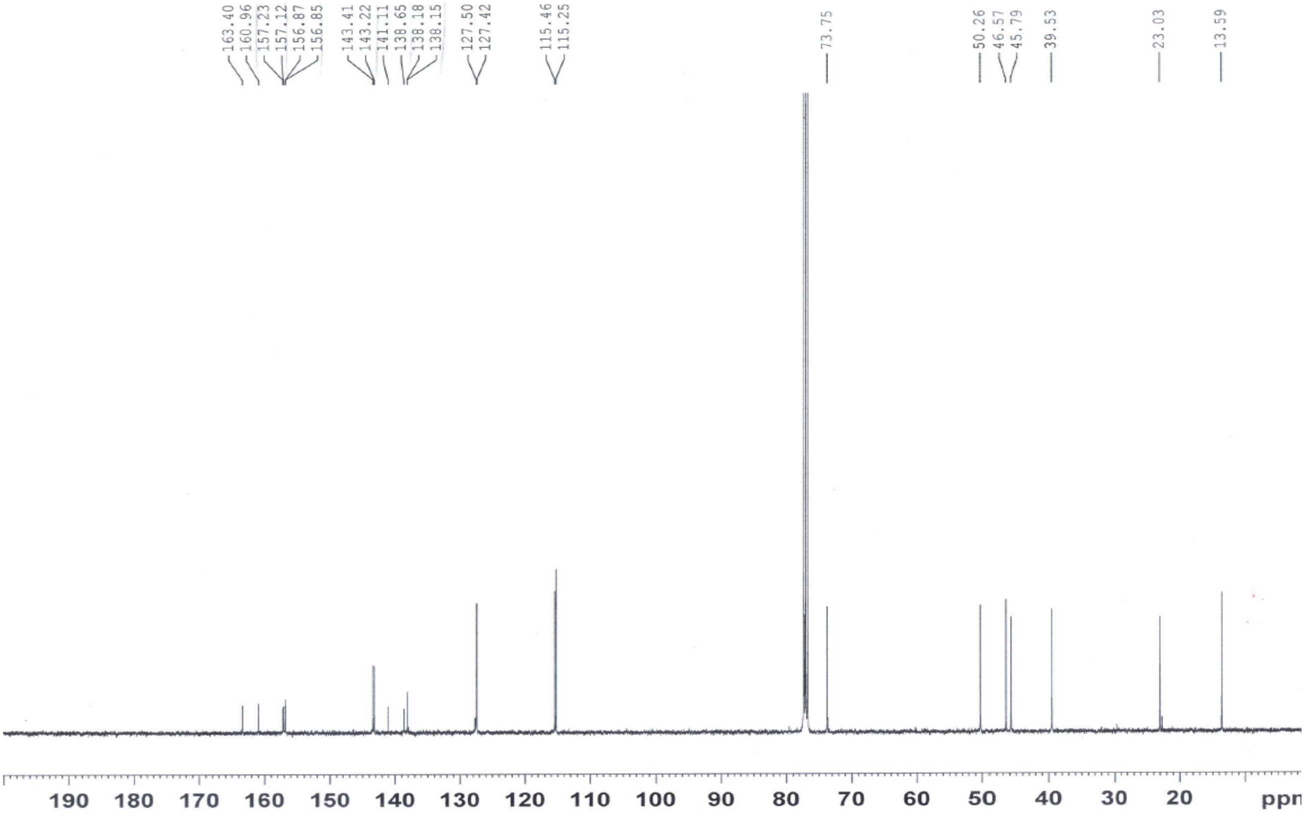

^{13}C NMR Spectrum of Compound (*R*,*R*)-**4e** (100 MHz)

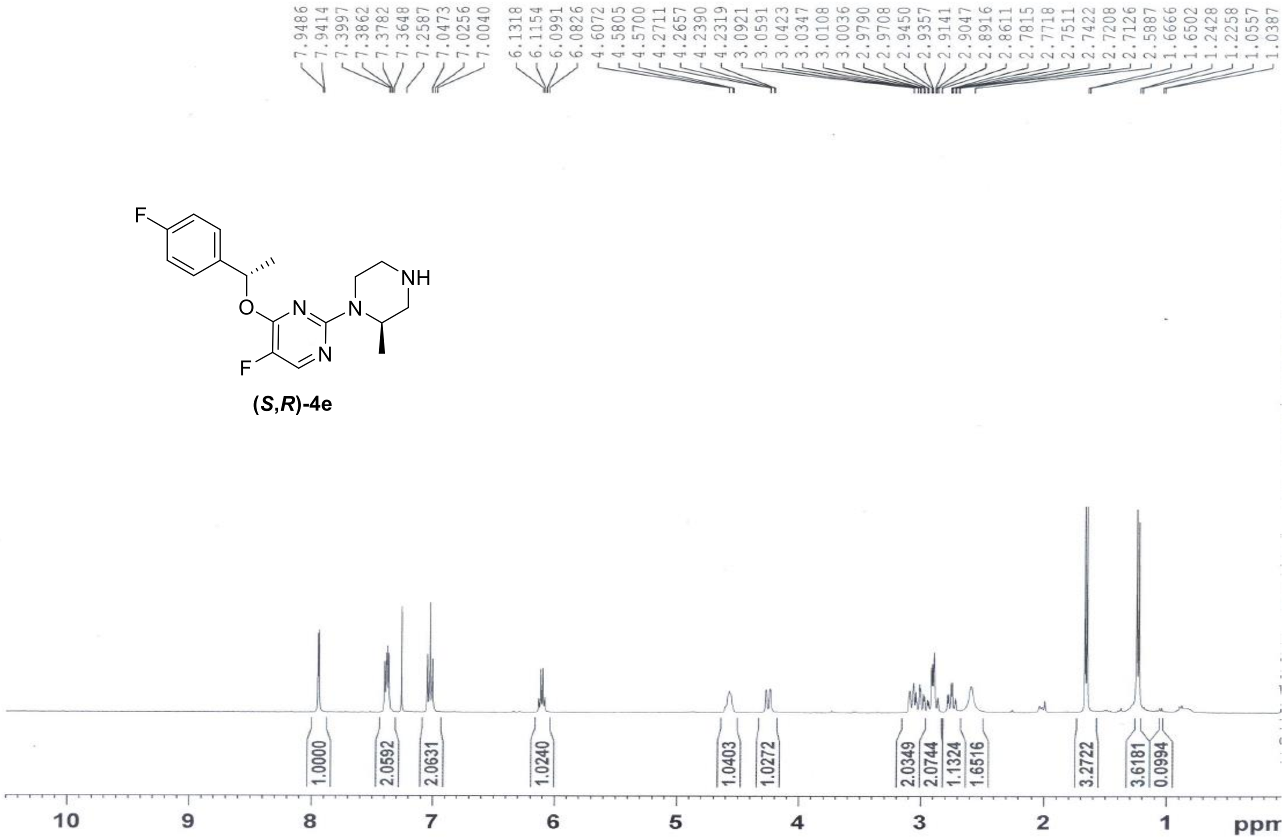

^{1}H NMR Spectrum of Compound (*S*,*R*)-**4e** (400 MHz)

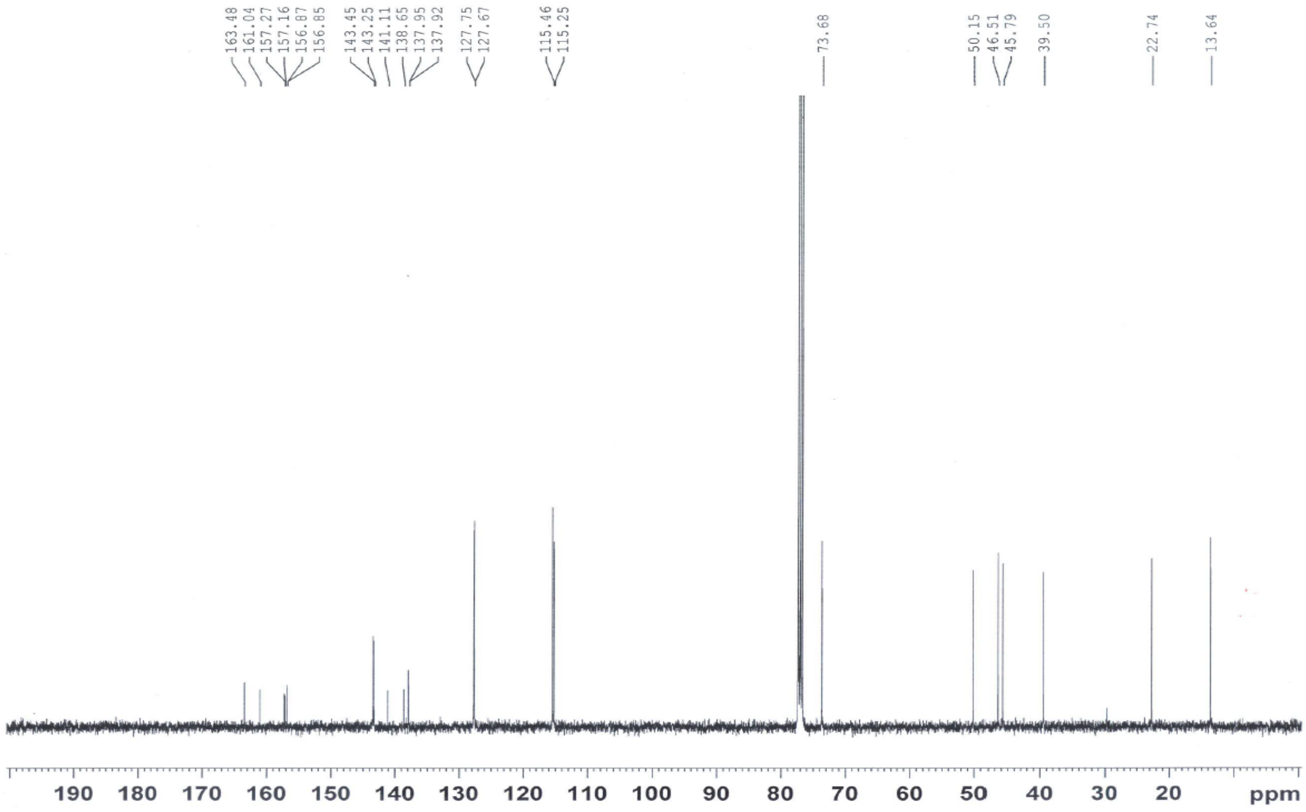

^{13}C NMR Spectrum of Compound (*S*,*R*)-**4e** (100 MHz)
